# Microarray gene expression profiling in colorectal (HCT116) and hepatocellular (HepG2) carcinoma cell lines treated with *Melicope ptelefolia* leaf extract reveals transcriptome profiles exhibiting anticancer activity

**DOI:** 10.7717/peerj.5203

**Published:** 2018-07-18

**Authors:** Mohammad Faujul Kabir, Johari Mohd Ali, Onn Haji Hashim

**Affiliations:** Department of Molecular Medicine, Faculty of Medicine, University of Malaya, Kuala Lumpur, Malaysia

**Keywords:** Melicope ptelefolia, HepG2, Hepatocellular carcinoma, Colorectal cancer, Ingenuity pathway analysis, Microarray, HCT116, Transcriptome profiling, Cancer

## Abstract

**Background:**

We have previously reported anticancer activities of *Melicope ptelefolia* (MP) leaf extracts on four different cancer cell lines. However, the underlying mechanisms of actions have yet to be deciphered. In the present study, the anticancer activity of MP hexane extract (MP-HX) on colorectal (HCT116) and hepatocellular carcinoma (HepG2) cell lines was characterized through microarray gene expression profiling.

**Methods:**

HCT116 and HepG2 cells were treated with MP-HX for 24 hr. Total RNA was extracted from the cells and used for transcriptome profiling using Applied Biosystem GeneChip™ Human Gene 2.0 ST Array. Gene expression data was analysed using an Applied Biosystems Expression Console and Transcriptome Analysis Console software. Pathway enrichment analyses was performed using Ingenuity Pathway Analysis (IPA) software. The microarray data was validated by profiling the expression of 17 genes through quantitative reverse transcription PCR (RT-qPCR).

**Results:**

MP-HX induced differential expression of 1,290 and 1,325 genes in HCT116 and HepG2 cells, respectively (microarray data fold change, MA_FC ≥ ±2.0). The direction of gene expression change for the 17 genes assayed through RT-qPCR agree with the microarray data. In both cell lines, MP-HX modulated the expression of many genes in directions that support antiproliferative activity. IPA software analyses revealed MP-HX modulated canonical pathways, networks and biological processes that are associated with cell cycle, DNA replication, cellular growth and cell proliferation. In both cell lines, upregulation of genes which promote apoptosis, cell cycle arrest and growth inhibition were observed, while genes that are typically overexpressed in diverse human cancers or those that promoted cell cycle progression, DNA replication and cellular proliferation were downregulated. Some of the genes upregulated by MP-HX include pro-apoptotic genes (DDIT3, BBC3, JUN), cell cycle arresting (CDKN1A, CDKN2B), growth arrest/repair (TP53, GADD45A) and metastasis suppression (NDRG1). MP-HX downregulated the expression of genes that could promote anti-apoptotic effect, cell cycle progression, tumor development and progression, which include BIRC5, CCNA2, CCNB1, CCNB2, CCNE2, CDK1/2/6, GINS2, HELLS, MCM2/10 PLK1, RRM2 and SKP2. It is interesting to note that all six top-ranked genes proposed to be cancer-associated (PLK1, MCM2, MCM3, MCM7, MCM10 and SKP2) were downregulated by MP-HX in both cell lines.

**Discussion:**

The present study showed that the anticancer activities of MP-HX are exerted through its actions on genes regulating apoptosis, cell proliferation, DNA replication and cell cycle progression. These findings further project the potential use of MP as a nutraceutical agent for cancer therapeutics.

## Introduction

Cancer is recognized by distinct abnormalities or dysregulations of genes, which cause sustained proliferative signaling, evasion of growth suppressors, activation of invasion and metastasis, replicative immortality, induction of angiogenesis and resisting cell death (Hanahan & Weinberg, 2011). An effective cancer drug should ideally be able to overcome most of these dysregulations and achieve the desired therapeutic effect with minimal side effects (Pérez-Herrero & Fernández-Medarde, 2015).

The plant kingdom provides a plethora of novel molecules for numerous therapeutic uses, which include treatment and prevention of chronic diseases including cancer. The study of plant extracts bioactivity is an important step for drug discovery and development, as it could lead to isolation of novel bioactive phytochemical(s) with therapeutic and medicinal properties (Singh et al., 2016). Some examples of anticancer drugs isolated from the plant kingdom include colchicine, podophyllotoxin, vincristine, vinblastine and taxol (Singh et al., 2016). Vinblastine is an antimitotic agent that is clinically indicated in the treatment of patients with Hodgkin’s and non-Hodgkin’s lymphomas, breast cancer, Kaposi’s sarcoma, renal cell cancer, and testicular cancer (Prendergast & Jaffee, 2013).

*Melicope ptelefolia* (MP) is a well-known herb in several Asian countries, including Malaysia, Indonesia, Thailand and Vietnam. In Malaysia, MP is locally known as “tenggek burung” and commonly used in a vegetable salad. MP has been used as a traditional medicine in Malaysia to treat several illnesses including high blood pressure, fatigue and erectile dysfunction (Aman, 2006). We have recently reported the anticancer and apoptosis induction activities of MP on colorectal, breast and liver cancer cell lines. The hexane leaf extract (MP-HX) appeared to show the most notable anti-proliferative activity against the four cancer cell lines tested (Kabir et al., 2017). However, the underlying molecular mechanisms involved have yet to be fully elucidated. The aim of the present study was to characterize anticancer activity of MP-HX on colorectal HCT116 and hepatocellular HepG2 carcinoma cell lines through microarray gene expression profiling.

## Materials and Methods

### Extract preparation

Fresh, healthy and young MP leaves were purchased from the local wet market and processed on the same day. The sample identity was authenticated by a plant taxonomist at the University of Malaya herbarium, Dr. Sugumaran Manickam. A voucher specimen was also deposited at the herbarium, with a registration number KLU 49190. The leaves were washed with distilled water and air dried for 3 days at room temperature. Sample drying was completed by incubating the leaves in an oven at 40 °C for 24 h. The dried leaves were then powdered using a table blender and stored at –20 °C until further analysis. MP-HX extract preparation was initiated by mixing fifty grams of the powdered leaves with 500 mL of hexane (1:10 ratio of sample weight to solvent volume). The mixture was constantly shaken (150 rpm) for 6 h at 37 °C using Innova 4300 Incubator Shaker (New Brunswick Scientific). The mixture was centrifuged at 1,500 rpm for 10 min, after which the supernatant was collected and filtered using a Whatman filter paper (No. 4). The residues were extracted again with the same solvent twice. The hexane solvent collected (∼1,500 mL) was evaporated at 40 °C using a rotary evaporator (Buchi Rotavapor R-215). The dried extract was dissolved in 10% dimethyl sulfoxide (DMSO) at 2 mg/mL and stored at –20 °C.

### Cell culture

Human colorectal (HCT116) and hepatocellular (HepG2) carcinoma cell lines were purchased from American Type Culture Collection (ATCC) and were cultured in Dulbecco’s modified minimum essential media (DMEM) (Catalogue No. 08458-45, Nacalai Tesque), supplemented with 10% FBS (Catalogue No. 10270, Gibco), 100 U/mL penicillin and 100 µg/mL streptomycin (09367-34, Nacalai Tesque). Cells were cultured in a 37 °C incubator with 5% CO_2_.

### Summary of study workflow

**Figure 1:**
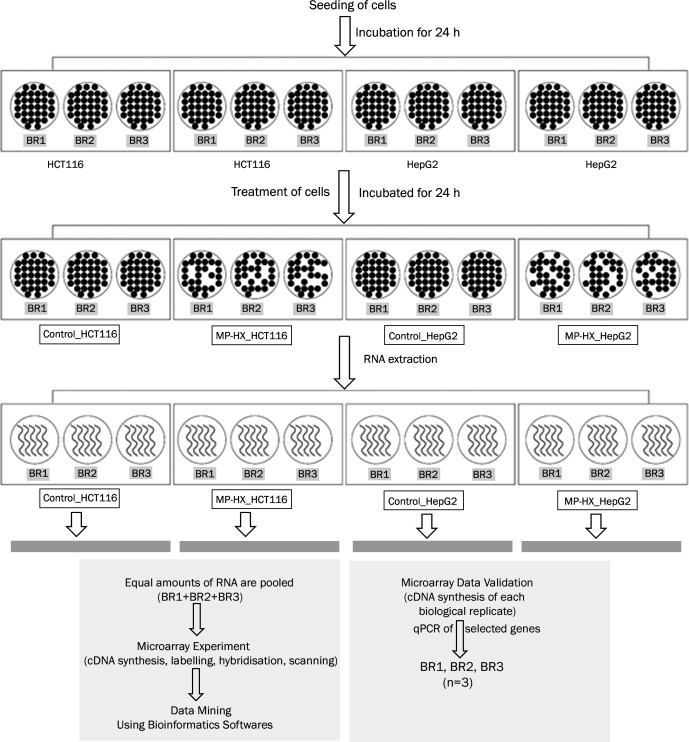
Workflow scheme for transcriptome profiling of HCT116 and HepG2 cells treated with MP-HX. The figure shows the workflow summary employed for the present study. BR, biological replicate.

The workflow employed for the present study is summarized in Fig. 1. The workflow involves seeding of HCT116 and HepG2 cells, treatment of the cells with either MP-HX or 0.39% DMSO (vehicle control), extraction of RNA, cDNA synthesis & labelling, microarray hybridization and scanning, validation of microarray data through quantitative reverse transcription PCR (RT-qPCR) and bioinformatics analysis of the dataset. The experimental protocols are detailed in the next sections.

### RNA extraction

For RNA extraction, the cells were seeded in 6 wells plate and incubated for 24 h. Following that, the cells were treated for 24 h with MP-HX at ∼IC_50_ concentration of the cell lines, which was 60 µg/mL for HCT116 and 78 µg/mL for HepG2 (Kabir et al., 2017). The control cells were treated with the vehicle (DMSO) used to dissolve MP-HX, and the final concentration of DMSO was 0.39 % v/v. After 24-hour treatment, total RNA from HCT116 and HepG2 cells were extracted and purified using PureLink RNA mini kit (Catalogue No. 12183018A; Invitrogen, Carlsbad, CA, USA), according to manufacturer’s instructions. RNA quantity and purity were verified using Nanodrop™ 2000 (Thermo Fisher Scientific, Waltham, MA, USA), by measuring absorbance ratios at 260/280 and 260/230. The RNA quality and integrity were also verified through agarose gel electrophoresis. Only RNA of good quality and integrity with an absorbance ratio of at least 2.0 was used for microarray and RT-qPCR assays.

### Microarray gene expression profiling

Microarray profiling was performed using Human Gene (HuGene) 2.0 ST array (Catalogue No. 902499; Applied Biosystems, Foster City, CA, USA). Briefly, equal amounts of total RNA from three biological replicates of an assay point were pooled to get a minimum of 500 ng total RNA (Ruiz-Laguna et al., 2016). The RNA was then reverse transcribed to synthesize complementary RNA (cRNA) using GeneChip™ WT Plus kit (Catalogue No. 902280; Applied Biosystems). The purified cRNA was quantified using Nanodrop™ 2000. Following this, cDNA was synthesized from 15 µg of cRNA. The purified cDNA (5.5 µg) was fragmented, labelled and hybridized onto GeneChip™ Human Gene 2.0 ST array, according to manufacturer’s guidelines. After 16 h of hybridisation in GeneChip™ Hybridization Oven 645, the arrays were washed and stained using GeneChip™ Expression, Wash and Stain kit (Catalogue No. 900720; Applied Biosystems) on GeneChip™ Fluidics Station 450. The stained arrays were scanned using GeneChip™ Scanner 3000 7G. Raw data (CEL files) was generated using GeneChip Operating Software (GCOS). The CEL files were analyzed by Expression Console software (version 1.4) (Applied Biosystems), to ensure the quality control parameters and the data produced by the arrays were acceptable. Gene expression data was analyzed using Transcriptome Analysis Console (TAC) software (version 3.1) (Applied Biosystems). The genes were filtered using microarray fold change (MA_FC) value of ±2.00. The list of genes (MA_FC ≥ ±2.00) was also analyzed using Ingenuity Pathway Analysis (IPA) (http://www.ingenuity.com) software to identify the canonical pathways, biological functions, gene networks and key processes that were enriched/modulated by MP-HX in HCT116 and HepG2 cell lines. The raw microarray data files were submitted to Gene Expression Omnibus (GEO) repository (accession number GSE114743).

### Quantitative reverse transcription PCR (RT-qPCR)

Each data point presented for the quantitative PCR assay was derived from three biological replicates (BR). Total RNA from each BR (Fig. 1) was reversed transcribed and the cDNA from each BR was used as a template for the qPCR. One microgram of total RNA was converted to cDNA using Maxima First Strand cDNA synthesis kit (Catalogue No. K1641; Thermo Fisher Scientific). The RT-qPCR was performed using Luminaris color HiGreeen High ROX qPCR master mix (Catalogue No. K0362; Thermo Fisher Scientific) on StepOne Plus PCR thermocycler (Applied Biosystems). Seventeen genes were selected to validate the microarray data, comprising of nine upregulated genes (BBC3, CDKN1A, CDKN2B, DDIT3, GABARAPL1, GADD45A, JUN, NDRG1, TP53) and eight downregulated genes (CCNA2, GINS2, HELLS, MCM2, MCM10, PLK1, RRM2 and SKP2). The primers used in the assay are listed in Table 1. The thermocycling condition for the real-time PCR was 50 °C for 2 min, 95 °C for 10 min, followed by 40 cycles of 95 °C for 15s, 60 °C for 30s (except for MCM2 and CDKN2B, 63 °C) and extension at 72 °C for 30s. RPS29 was selected as the internal control gene to normalize the gene expression data (De Jonge et al., 2007). Relative quantification of the target genes was calculated by comparative 2^−ΔΔ*CT*^ method (Livak & Schmittgen, 2001).

**Table 1:** Oligonucleotide sequences used for real-time qPCR assay.

Gene name	Forward (5′-3′)	Reverse (5′-3′)
BBC3	cccgtgaagagcaaatgag	accccctgatgaaggtgag
CDKN1A	gaccatgtggacctgtcac	ggcttcctcttggagaagatc
CDKN2B	atcccaacggagtcaaccg	agtctcagacaggcttgcagg
DDIT3	tcaccacacctgaaagcag	gagccgttcattctcttcag
GABARAPL1	gaccatccctttgagtatcgg	gaagaataaggcgtcctcaggtc
GADD45A	gttttgctgcgagaacgac	gaacccattgatccatgtag
JUN	ttctatgacgatgccctcaacgcctc	gaagccctcctgctcatctgtcacgttc
NDRG1	gcagagtaacgtggaagtggtcc	acggcatccactgcaggc
TP53	tgactgtaccaccatccactacaac	ttgcggagattctcttcctctg
CCNA2	cagaaaaccattggtccctc	cactcactggcttttcatcttc
GINS2	gccgagaaggagctggttac	gtttcaggttaatcgccagc
HELLS	gcttgatgggtccatgtctt	gacatctatcctgggcctga
MCM2	aagtgcaatttcgtcctgggtc	gcaaccttgttgtccttcttgg
MCM10	gcaaaaatcccctgtagagaag	tccccacaatttgacctctag
PLK1	acttcgtgttcgtggtgttgg	gcttgaggtctcgatgaataac
RRM2	gcgatttagccaagaagttcag	cccagtctgccttcttcttg
SKP2	cgctgcccacgatcatttat	ggagattctttctgtagccgct
RPS29	gcactgctgagagcaagatg	ataggcagtgccaaggaaga

### Statistical analysis

Statistical comparisons were carried out by independent *t*-test using SPSS statistical software version 22.0 (SPSS Inc., Chicago, IL, USA). Values are shown as mean ±SD. The acceptable level for statistical significance was *P* < 0.05.

## Results

### Microarray data validation by RT-qPCR

The validation was performed by measuring the expression of 17 different genes (nine upregulated, eight downregulated) through RT-qPCR. These genes were selected based on their important roles in processes such as cell cycle progression, cellular proliferation and programmed cell death. The selected genes had microarray fold change (MA_FC) value of ≥ ± 1.50, except TP53, which had MA_FC of approximately +1.30. Their differential expression likely made significant contribution towards MP-HX anticancer activity. A comparison of FC values between microarray (MA_FC) and RT-qPCR (qPCR_FC) techniques are shown in Table 2 and Fig. S1. The result showed that the direction of gene expression changes as revealed by RT-qPCR assay (17/17) was in agreement with the microarray data.

**Table 2:** Comparison of gene expression fold changes (FCs) obtained from microarray (MA_FC) and real-time qPCR (qPCR_FC) assays. The table shows gene expression FCs obtained by MA and qPCR assays. For qPCR assay, the data were normalized to RPS29 expression and each value represents mean qPCR_FC  ± SD (*n* = 3). A positive FC value indicates upregulation, while negative FC value indicates downregulation.

Cell line	Gene symbol	Gene name	MA_FC	qPCR_FC
HCT116	1. BBC3	BCL2 binding component 3	+2.93	+4.48 ± 0.69
	2. CDKN1A	Cyclin-dependent kinase inhibitor 1A	+2.36	+4.44 ± 0.33
	3. DDIT3	DNA-damage-inducible transcript 3	+8.36	+12.29 ± 0.15
	4. GADD45A	Growth arrest and DNA-damage-inducible, alpha	+3.23	+5.49 ± 0.49
	5. NDRG1	N-myc downstream regulated 1	+3.16	+6.52 ± 0.83
	6. GABARAPL1	GABA(A) receptor-associated protein like 1	+3.74	+8.35 ± 0.63
	7. JUN	Jun proto-oncogene	+4.53	+9.45 ± 0.86
	8. CDKN2B	Cyclin-dependent kinase inhibitor 2B	+3.95	+9.82 ± 0.21
	9. TP53	Tumor protein p53	+1.38	+2.01 ± 0.04
	10. CCNA2	Cyclin A2	−4.21	−18.89 ± 1.92
	11. GINS2	GINS complex subunit 2 (Psf2 homolog)	−7.03	−6.73 ± 0.10
	12. HELLS	Helicase, lymphoid-specific	−6.15	−6.43 ± 0.27
	13. RRM2	Ribonucleotide reductase M2	−3.80	−7.45 ± 1.05
	14. PLK1	Polo-like kinase 1	−4.60	−19.52 ± 0.87
	15. SKP2	S-phase kinase-associated protein 2	−4.86	−10.90 ± 0.17
	16. MCM2	Minichromosome maintenance complex component 2	−4.70	−32.97 ± 2.84
	17. MCM10	Minichromosome maintenance 10 replication initiation factor	−5.86	−23.99 ± 3.78
HepG2	1. BBC3	BCL2 binding component 3	+2.71	+2.68 ± 0.38
	2. CDKN1A	Cyclin-dependent kinase inhibitor 1A	+2.31	+2.03 ± 0.14
	3. DDIT3	DNA-damage-inducible transcript 3	+5.21	+7.69 ± 0.24
	4. GADD45A	Growth arrest and DNA-damage-inducible, alpha	+2.48	+2.12 ± 0.07
	5. NDRG1	N-myc downstream regulated 1	+2.29	+5.69 ± 1.70
	6. GABARAPL1	GABA(A) receptor-associated protein like 1	+4.87	+5.63 ± 0.14
	7. JUN	Jun proto-oncogene	+5.06	+6.00 ± 1.04
	8. CDKN2B	Cyclin-dependent kinase inhibitor 2B	+2.30	+3.52 ± 0.59
	9. TP53	Tumor protein p53	+1.34	+1.41 ± 0.05
	10. CCNA2	Cyclin A2	−2.66	−6.18 ± 0.52
	11. GINS2	GINS complex subunit 2 (Psf2 homolog)	−2.37	−2.19 ± 0.07
	12. HELLS	Helicase, lymphoid-specific	−4.03	−3.01 ± 0.14
	13. RRM2	Ribonucleotide reductase M2	−2.19	−3.83 ± 0.73
	14. PLK1	Polo-like kinase 1	−1.87	−14.94 ± 2.33
	15. SKP2	S-phase kinase-associated protein 2	−3.65	−28.53 ± 3.72
	16. MCM2	Minichromosome maintenance complex component 2	−1.59	−12.90 ± 2.53
	17. MCM10	Minichromosome maintenance 10 replication initiation factor	−4.04	−21.03 ± 1.00

### Analysis of microarray data using Transcriptome Analysis Console (TAC)

**Figure 2:**
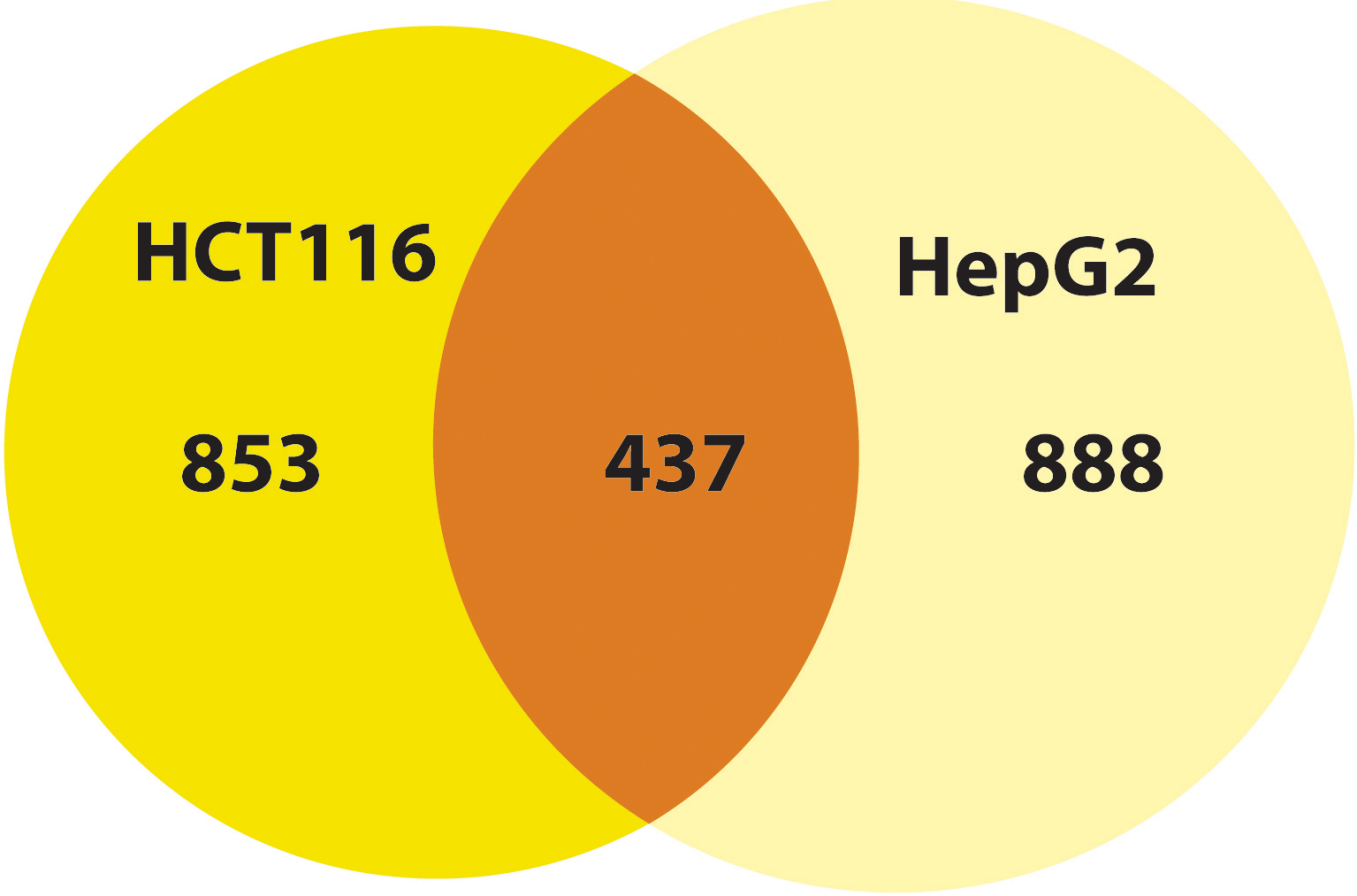
MP-HX treatment induced differential expression of genes inHCT116 and HepG2 cells. The Venn diagram shows the number of differentially expressed genes induced by MP-HX in HCT116 and HepG2 cells (MA_FC ≥ ±2.00).

Using a cut-off fold change (MA_FC) value of ±2.00, 1,290 and 1,325 genes were noted to be differentially regulated in HCT116 and HepG2 cells, respectively. Among these genes, 437 were differentially regulated in both cell lines (Fig. 2). Subjecting the genes to TAC software analysis revealed a total of 130 and 164 Wikipathways (WPs) that were significantly regulated (*p* < 0.05) in HCT116 and HepG2 cells, respectively (Data S1). TAC ranked the WPs based on significance score.

Among the WPs that were significantly regulated, “Retinoblastoma (RB) in cancer” (RIC-WP) ranked the highest in HCT116 and HepG2 cells, with significant scores of 36.09 and 22.82, respectively. In both cell lines, almost all of RIC-WP genes which can promote cell cycle progression and proliferation were downregulated. This pathway interacted with 45 genes (one upregulated, 44 downregulated) in HCT116 cells, and 35 genes (four upregulated, 31 downregulated) in HepG2 cells (Figs. S2 and S3). These figures showed that MP-HX treatment induced downregulation of E2F, CDK1, CDK2, CDK6, CCNA2, CCNB1, CCNB2, CCNE1 and CCNE2 in both cell lines.

The ‘G_1_ to S cell cycle control’ Wikipathway (G_1_SCC-WP) had significant scores of 18.42 in HCT116 and 10.58 in HepG2 cells (Figs. S4 and S5). Twenty-seven genes in G_1_SCC-WP were modulated in HCT116 cells, of which 23 were downregulated. In HepG2 cells, 20 genes were modulated, of which 15 were downregulated. The G_1_/S cell cycle transition is activated by cyclins, CDKs and E2F proteins. This transition can be inhibited by CDK inhibitors. From the figures, many genes in G_1_SCC-WP which can promote proliferation were downregulated in both cell lines (CDC25A, CCNE2, PCNA, MCM3/5/6, POLE2, POLA2, CDK6, PRIM1, CDK1, CCNB1). In contrast, MP-HX upregulated GADD45A, CDK inhibitors p15 (CDKN2B) and p21 (CDKN1A) in both cell lines (Figs. S4 and S5).

The ‘Cell Cycle’ WikiPathway (CC-WP) (Figs. S6 and S7) had significant scores of 17.05 in HCT116 and 7.93 in HepG2. In HCT116, 31 genes were modulated, and they were all downregulated. In contrast, HepG2 cells showed modulation of 21 genes, of which 19 were downregulated and only two were upregulated. The dataset showed that MP-HX downregulated the expression of many cell cycle promoting genes in CC-WP in both cell lines, and these include CCNA2, CCNB1, CCNB2, CCNE2, CDK1, CDK2 and CDK6 (Figs. S6 and S7). MP-HX also downregulated MCM2 and PLK1 expression in HCT116, while MCM10 was downregulated in both cell lines.

The “DNA damage response” WikiPathway (DDR-WP) was also modulated by MP-HX, with significance score values of 6.67 and 6.38 in HCT116 and HepG2, respectively. In HCT116, four genes were upregulated and 11 genes were downregulated (Fig. S8). In HepG2, six genes were upregulated and nine genes were downregulated (Fig. S9). MP-HX significantly downregulated CHEK1, RAD51, CDC25A, cyclins and CDKs expression, while CDKN1A, BBC3 and GADD45A were upregulated in both cell lines.

The “Apoptosis” Wikipathway (AP-WP) is was modulated by MP-HX with **s**ignificance scores of 3.95 and 3.16 in HCT116 and HepG2 cells, respectively (Figs. S10 and S11). In HCT116, 10 genes were upregulated and three genes were downregulated. In HepG2, 12 genes were modulated and seven of them were upregulated. MP-HX upregulated the expression of BBC3 and JUN while it downregulated the expression of HELLS in both cell lines. In HepG2 cells, BAK1 and PMAIP1 were also upregulated by MP-HX. On the other hand, MP-HX downregulated BIRC5 expression in HCT116.

### IPA analysis - Top canonical pathways modulated by MP-HX in HCT116 and HepG2 Cells

**Figure 3:**
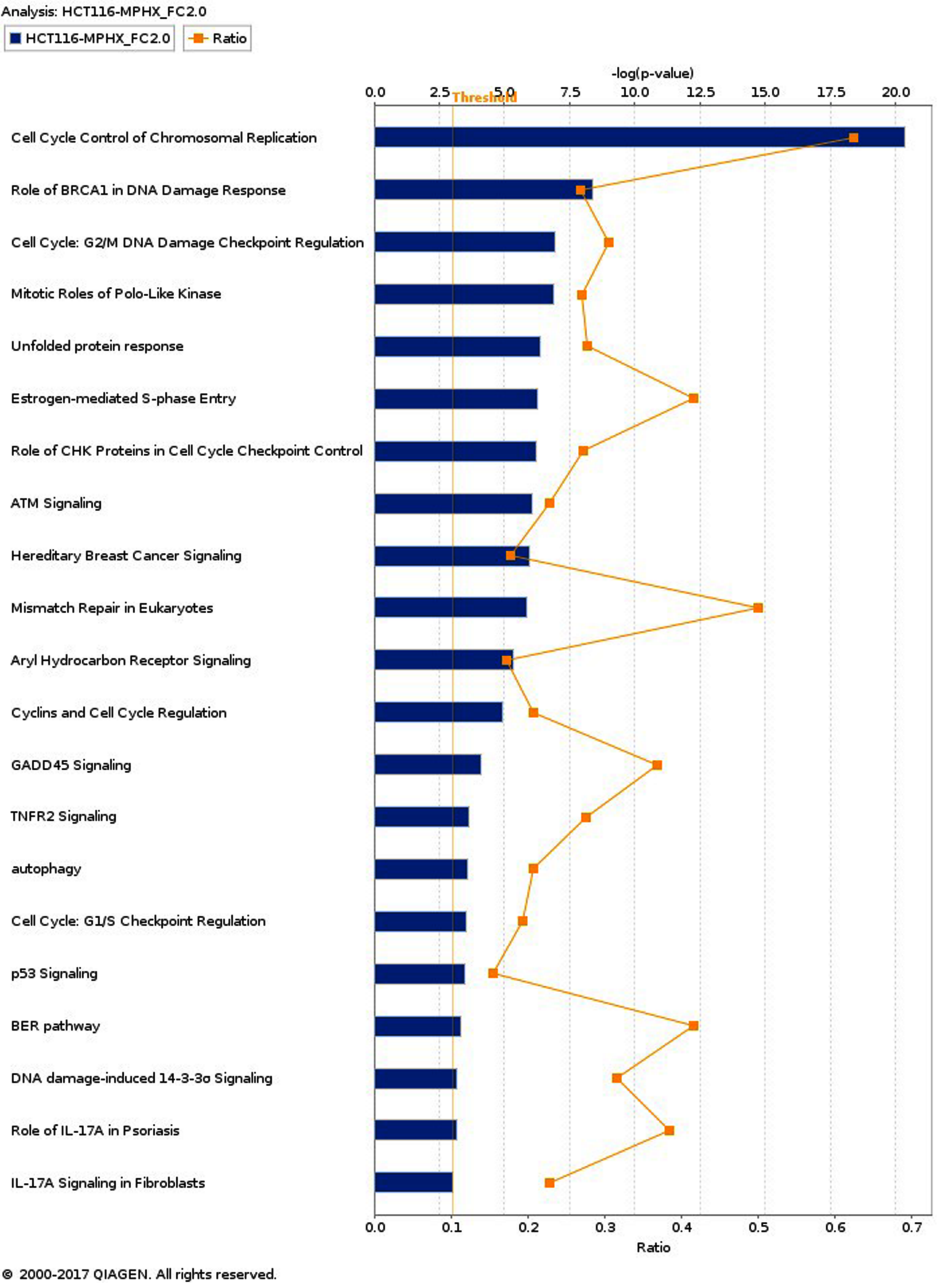
Top canonical pathways (CPs) enriched in HCT116 cells after MP-HX treatment. IPA software ranked the top CPs based on -log (*p*-value). Ratio value denotes the number of molecules in the dataset divided by the total number of molecules associated with the pathway. Data were analyzed through the use of IPA (QIAGEN Inc., https://www.qiagenbioinformatics.com/products/ingenuity-pathway-analysis).

**Figure 4:**
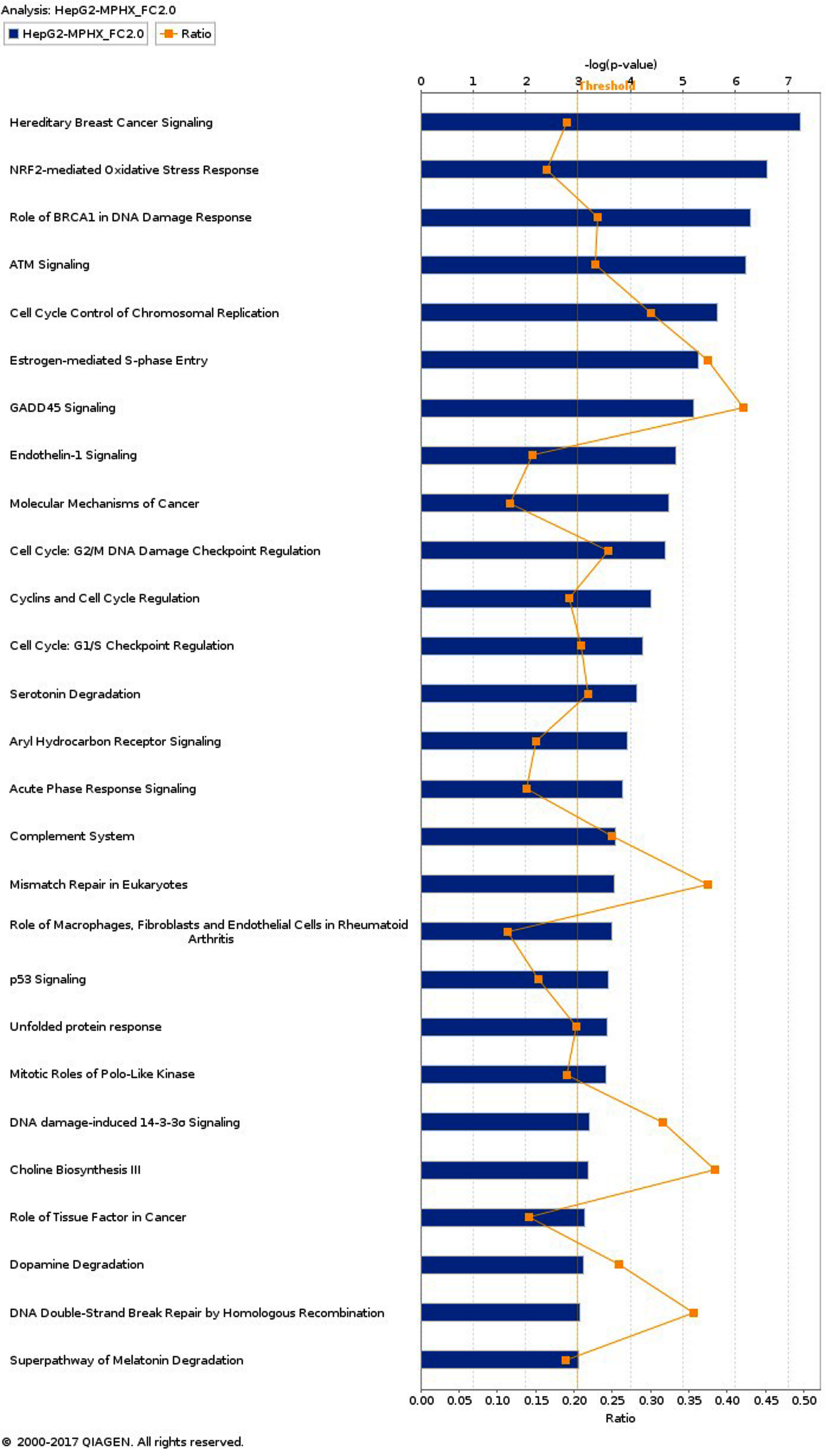
Top canonical pathways (CPs) enriched in HepG2 cells after MP-HX treatment. IPA software ranked the top CPs based on -log (*p*-value). Ratio value denotes the number of molecules in the dataset divided by the total number of molecules associated with the pathway. Data were analyzed through the use of IPA (QIAGEN Inc., https://www.qiagenbioinformatics.com/products/ingenuity-pathway-analysis).

IPA enriched 55 and 135 canonical pathways (*p* < 0.05) in HCT116 and HepG2, respectively. Canonical pathways (CPs) with -log (*p*-value) of 3.0 and above are shown in Figs. 3 and  4. In HCT116, top most significant CPs include “cell cycle control of chromosomal replication”, “role of BRCA1 in DNA damage response”, “cell cycle: G_2_/M DNA damage checkpoint regulation”, “mitotic roles of polo-like kinase”, “unfolded protein response”, and “estrogen mediated S-phase entry” (Fig. 3). In HepG2, top CPs were “hereditary breast cancer signaling”, “NRF2- mediated oxidative stress response”, “role of BRCA1 in DNA damage response”, “ATM signaling”, “cell cycle control of chromosomal replication”, “estrogen mediated S-phase entry” and “GADD45 signaling” (Fig. 4).

A comparison of over-represented CPs in HCT116 and HepG2 are shown in Table 3. The CPs are ranked according to their respective -log (*p*-value). Based on the list, HCT116 showed a higher –log (*p*-value) for most of the CPs. In HepG2, only three CPs showed higher –log (*p*-value) than that of HCT116, and these include “hereditary breast cancer signaling”, “ATM signaling” and “NRF2-mediated oxidative stress response”. These observations could mean that although MP-HX may have exerted a similar anticancer effect in both cell lines, a subset of different effect or mechanism of action may have also occurred between the cell lines.

**Table 3:** Modulation of canonical pathways (CPs) in HCT116 and HepG2 by MP-HX. The table shows the top canonical pathways modulated by MP-HX in the cell lines. They were ranked by IPA software based on −log (*p*-value).

Canonical pathway	−log (*p*-value)	
	HCT116	HepG2
1. Cell Cycle Control of Chromosomal Replication	20.34	5.67
2. Role of BRCA1 in DNA Damage Response	8.36	6.28
3. Hereditary Breast Cancer Signaling	5.95	7.25
4. ATM Signaling	6.08	6.19
5. Cell Cycle: G2/M DNA Damage Checkpoint Regulation	6.96	4.66
6. Estrogen-mediated S-phase Entry	6.25	5.29
7. Mitotic Roles of Polo-Like Kinase	6.91	3.54
8. Unfolded protein response	6.35	3.55
9. NRF2-mediated Oxidative Stress Response	2.96	6.61
10. Mismatch Repair in Eukaryotes	5.84	3.68

### IPA analysis - Diseases and Functions Modulated by MP-HX in HCT116 and HepG2 Cells

The top category of diseases and functions modulated in HCT116 and HepG2 cells by MP-HX include “cancer”, “organismal injury and abnormalities”, “gastrointestinal disease”, “cell death and survival”, “cell cycle”, “cellular assembly and organization”, “DNA replication, recombination and repair”, “cellular development” and “cellular growth and proliferation” (Figs. S12 and S13). In both HCT116 and HepG2 cells, the predicted activation *z*-scores appeared supportive of MP-HX activity, as the negative values are suggestive of inhibition of processes associated with cell growth, cell proliferation and cell cycle (Table 4).

**Table 4:** Modulation of diseases and biological functions by MP-HX in HCT116 and HepG2 cells. The table shows the enriched terms corresponding to diseases or biological functions, along with the number of associated genes in the experimental dataset, *p*-value and activation *z*-score. The *z*-score is calculated by the IPA software which predicts whether a specific disease or bio-function is increased (positive *z*-score) or decreased (negative *z*-score) based on the experimental dataset.

Cell line	Categories	Diseases or biological functions	*p*-value	*z*-score	Number of genes
HCT116	Cellular development; cellular growth and proliferation	Cell proliferation of tumor cell lines	6.26E−16	−2.60	218
	DNA replication, recombination, and repair	DNA replication	3.48E−13	−2.07	27
	Cell cycle	S-phase	1.37E−11	−3.47	40
	Cell cycle	M-phase of tumor cell lines	5.02E−11	−2.00	28
	Cell cycle; DNA replication, recombination, and repair	Checkpoint control	6.93E−09	−2.55	15
	Cell death and survival	Cell death of cervical cancer cell lines	7.07E−08	+2.66	60
	Cell death and survival	Apoptosis of cervical cancer cell lines	1.71E−06	+3.37	47
	Cell cycle	G1-phase of tumor cell lines	4.41E−06	+2.39	35
	Cell cycle	Entry into interphase	7.87E−06	−2.30	17
	DNA replication, recombination, and repair	DNA damage	9.03E−06	+2.42	24
	Cell cycle	Entry into S-phase	1.42E−05	−2.57	16
	Cell death and survival	Cell viability of cervical cancer cell lines	3.01E−05	−2.18	34
	Cellular development; cellular growth and proliferation	Cell proliferation of breast cancer cell lines	3.58E−05	−2.94	61
HepG2	Cell death and survival	Apoptosis of cervical cancer cell lines	1.48E−08	+2.34	51
	Cell death and survival	Cell viability of cervical cancer cell lines	4.67E−05	−2.01	33
	Cell cycle	G1-phase of tumor cell lines	1.96E−04	+2.01	30
	Cell cycle	M-phase of tumor cell lines	2.17E−04	−2.34	17
	Cell cycle; DNA replication, recombination, and repair	Checkpoint control	5.56E−04	−2.38	9
	Cell death and survival; cellular development	Self-renewal of tumor cell lines	9.53E−04	−2.20	5

### IPA analysis - Upstream Regulators Prediction in HCT116 and HepG2 Cells

IPA upstream regulator analysis (URA) examines how many known targets of each transcriptional regulator (TR) are present in the dataset and compares their direction of change to what is expected from the literature to predict the likely relevant TRs. If the observed direction of change is mostly consistent with a particular activation state of the TR (“activated” or “inhibited”), then a prediction is computed about that activation state (*z*-score). This can provide insights on the underlying mechanism for the biological activities in the tissue/cells being studied.

**Figure 5:**
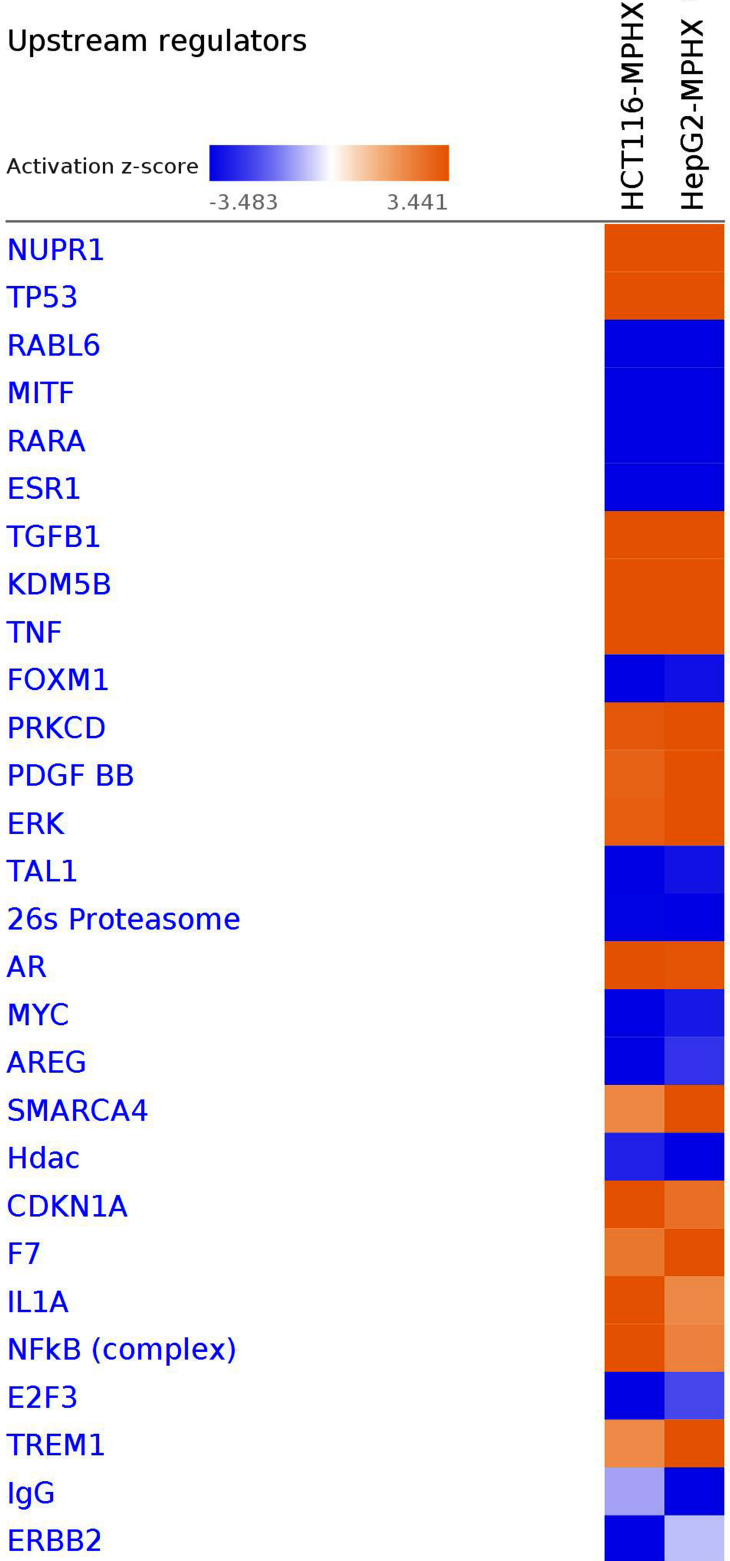
IPA software prediction of upstream regulators and their activation state in HCT116 and HepG2 cells after MP-HX treatment. IPA predicts modulation of regulators and ranked them based on the activation *z*-score. A positive score denotes activation, while a negative score denotes inhibition. The heatmap color intensity depicts relative value of activation *z*-score for the corresponding cell line. Upstream regulators were filtered based on -log (*p*-value) of 3.0 and activation *z*-score of 3.5. Orange color indicates activation whereas blue color indicates inhibition.

A comparison of top upstream regulators modulated in HCT116 and HepG2 cells is depicted in Fig. 5. The heatmap shows that most of the upstream regulators were modulated in the same direction in both cell lines, except IgG and ERBB2. There were only slight differences in the activation *z*-scores of AREG, SMARCA4, HDAC, CDKN1A, F7, IL1A, NFKB, E2F3 and TREM1 between the HCT116 and HepG2 cells. This observation suggests MP-HX exerted its effect by modulating a similar set of upstream regulators in both cell lines. The top upstream regulators that were predicted to be modulated by MP-HX in HCT116 and HepG2 cells are also listed in Table 5. NUPR1 was the most significant upstream regulator in HCT116 and HepG2 with activation *z*-scores of +10.94 and +12.21, respectively. IPA also ranked TP53 as the second most significant upstream regulator in both cell lines, with activation *z*-scores of +7.16 and +6.22 in HCT116 and HepG2 cells, respectively. Another upstream regulator predicted by IPA was RABL6 (RAB, member RAS oncogene family like 6), which had activation *z*-scores of −6.25 and −4.80 in HCT116 and HepG2 cells, respectively.

**Table 5:** IPA software prediction of upstream regulators in HCT116 and HepG2 cells that were treated with MP-HX. IPA predicts modulation of upstream regulators and ranked them based on the activation *z*-score. A positive score denotes activation, while a negative score denotes inhibition.

Cell line	Activation/Inhibition	UR	MA_FC	*z*-score	Overlap *p*-value
HCT116	Activated	NUPR1	+1.27	+10.94	8.34E−57
		TP53	+1.38	+7.16	3.07E−32
		TNF	+1.07	+4.81	1.15E−12
		KDM5B	+2.44	+4.79	9.05E−18
		IL1A	+2.74	+3.86	1.39E−08
		NFkB (complex)		+3.65	2.24E−12
		AR	+1.14	+3.61	4.8E−15
		CDKN1A	+2.36	+3.59	3.36E−10
		TGFB1	−1.20	+3.54	2.48E−21
		E2F6	+1.27	+3.46	1.01E−10
	Inhibited	RABL6	−1.28	−6.25	9.25E−37
		MITF	+1.12	−5.62	3.56E−26
		RARA	−1.24	−5.10	4.43E−11
		ESR1	−1.16	−4.91	9.74E−17
		FOXM1	−2.96	−4.89	2.09E−21
		TAL1	−1.21	−4.15	6.83E−08
		E2F3	−1.35	−3.71	1.99E−14
		MYC	−1.40	−3.67	3.23E−14
		ERBB2	−1.12	−3.54	7.77E−57
		E2f		−3.46	4.87E−19
HepG2	Activated	NUPR1	+2.40	+12.21	1.95E−73
		TP53	+1.34	+6.22	2.45E−27
		TGFB1	+2.29	+5.37	3.98E−17
		PDGF BB		+4.81	2.03E−14
		PRKCD	+1.63	+4.74	9.17E−11
		ERK		+4.68	3.94E−10
		SMARCA4	+1.06	+4.26	2.52E−10
		TREM1	+8.43	+3.75	1.56E−08
		TGM2	−1.34	+3.72	0.0044
		KDM5B	+1.08	+3.54	1.54E−13
	Inhibited	RABL6	−1.11	−4.80	1.15E−24
		RARA	+1.09	−4.40	1.22E−10
		MITF	+1.24	−4.19	7.93E−11
		ESR1	+1.08	−4.04	5.29E−17
		IgG		−3.84	6.49E−07
		26s Proteasome		−3.64	5.77E−14
		Hdac		−3.57	2.64E−08
		KIAA1524	−2.21	−3.26	7.02E−04
		FOXM1	−1.87	−3.20	6.48E−13
		TAL1	−1.22	−3.15	1.66E−06

### IPA analysis—Networks Enriched in HCT116 and HepG2 by MP-HX

IPA network analysis display the interaction between molecules present in the dataset which reflects significance of biological function. The IPA software ranked top networks enriched in the microarray dataset based on ‘network score’ Table 6. This analysis considers the number of network eligible molecules in the network, network size, total number of network eligible molecules analyzed and total number of network eligible molecules in the Ingenuity^®^ Knowledge Base that could be included in the network. The network score is calculated based on right tailed Fisher’s exact test and the presented network score is negative logarithm of Fisher’s exact *p*-value. The higher the score, the more significant is the interaction between network eligible molecules. The full list of molecules in the network indicated is listed in Data S2.

**Table 6:** Modulation of networks in HCT116 and HepG2 cells by MP-HX. IPA software ranked top networks enriched in the microarray dataset based on the network score (NS).

Cell line	NI	Score	Focus mlecules	Top disease and functions
HCT116	1	124	124	Cell Cycle, Cellular Assembly and Organization, DNA Replication, Recombination, and Repair
	2	114	119	Cellular Compromise, Cellular Function and Maintenance, Cancer
	3	73	94	Cell Death and Survival, Inflammatory Response, Cellular Development
	4	66	84	Cell Cycle, DNA Replication, Recombination, and Repair, Cell Death and Survival
	5	57	83	Cancer, Organismal Injury and Abnormalities, Respiratory Disease
HepG2	1	122	122	Cell Cycle, Cellular Assembly and Organization, DNA Replication, Recombination, and Repair
	2	110	116	Cell Death and Survival, Cell Morphology, Cellular Function and Maintenance
	3	68	90	Cell Death and Survival, Organismal Injury and Abnormalities, Cell Cycle
	4	61	85	Cell Cycle, Cancer, Organismal Injury and Abnormalities
	5	57	82	Cell Cycle, Cellular Assembly and Organization, DNA Replication, Recombination, and Repair

**Figure 6:**
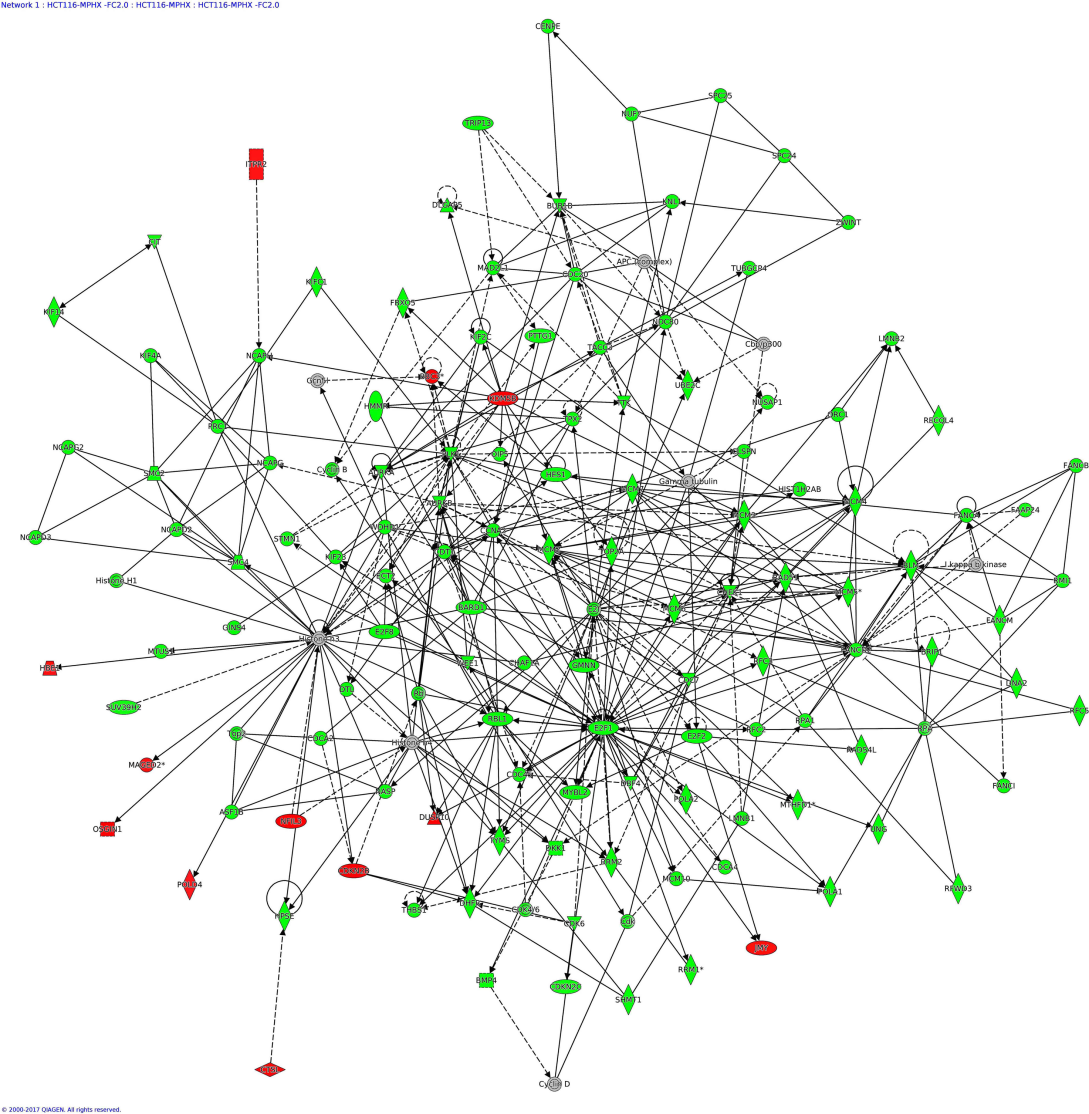
IPA network analysis: annotated interactions between genes in HCT116 cells treated with MP-HX. The figure shows network 1 in HCT116, containing 124 focus molecules. Most of the focus molecules in this network were downregulated by MP-HX. Top diseases and functions associated with this network include processes related to cell proliferation, cell cycle and DNA replication. Molecules in the network are colored red (MA_FC ≥  + 2.0), green (MA_FC ≥  − 2.0), or grey (MA_FC <  ± 2). Data were analyzed through the use of IPA (QIAGEN Inc., https://www.qiagenbioinformatics.com/products/ingenuity-pathway-analysis).

**Figure 7:**
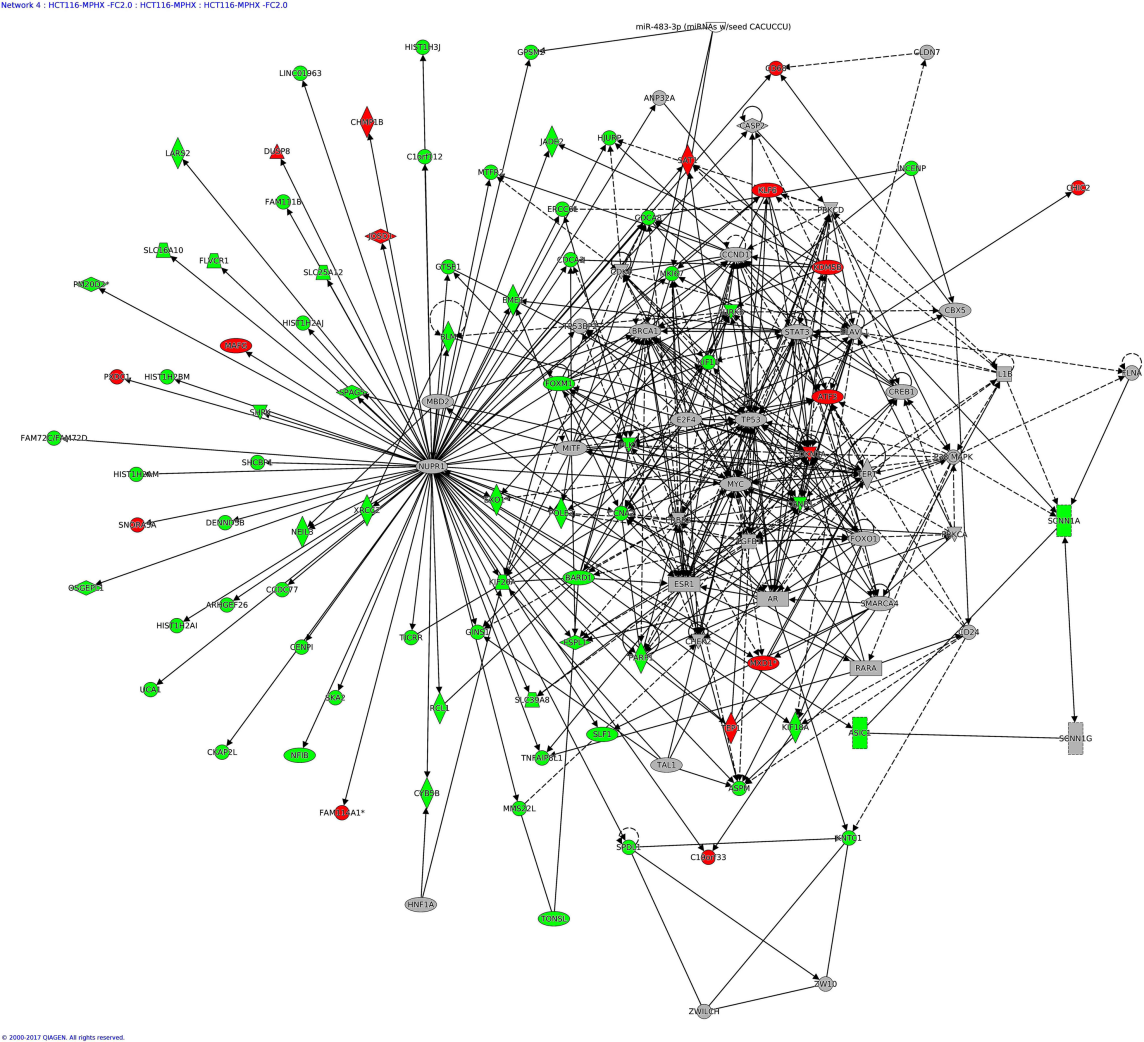
IPA network analysis: annotated interactions between genes in HCT116 cells treated with MP-HX. The figure shows network 4 in HCT116, containing 84 focus molecules. NUPR1 shows interaction with many other focus molecules that show expression downregulation (MA_FC ≥  − 2.0). Top diseases and functions associated with this network include processes related to cell cycle regulation, DNA replication and cell death/survival. Molecules in the network are colored red (MA_FC ≥  + 2.0), green (MA_FC ≥  − 2.0), or grey (MA_FC <  ± 2). Data were analyzed through the use of IPA (QIAGEN Inc., https://www.qiagenbioinformatics.com/products/ingenuity-pathway-analysis).

**Figure 8:**
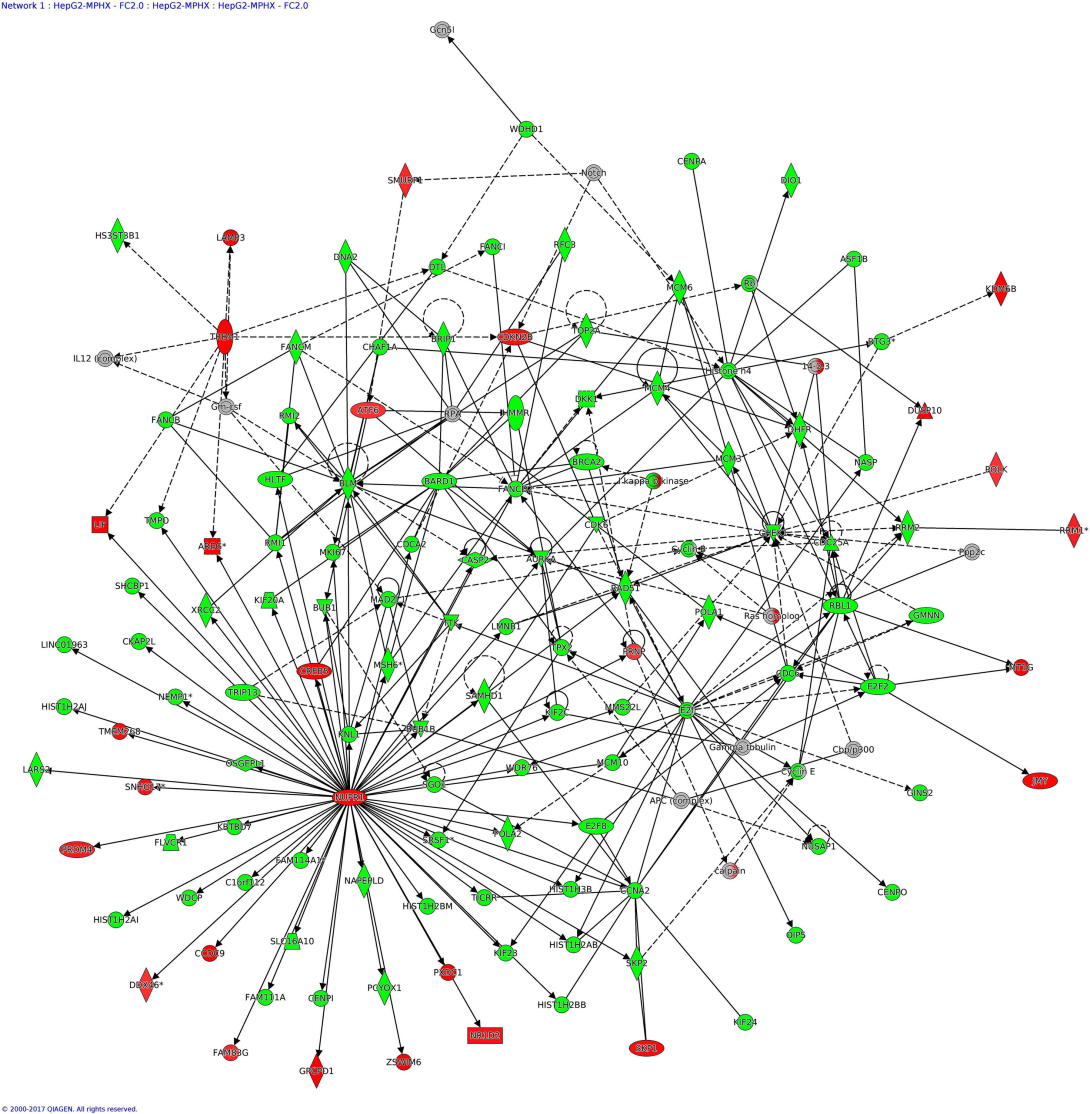
IPA network analysis: annotated interactions between genes in HepG2 cells treated with MP-HX. The figure shows network 1 in HepG2 cells, containing 122 focus molecules. NUPR1 (MA_FC +2.40) shows interaction with many other focus molecules that show expression downregulation (MA_FC ≥ − 2.0). Top diseases and functions associated with this network include processes related to cell cycle, cancer and DNA replication. Molecules in the network are colored red (MA_FC ≥ + 2.0), green (MA_FC ≥ − 2.0), or grey (MA_FC < ±2). Data were analyzed through the use of IPA (QIAGEN Inc., https://www.qiagenbioinformatics.com/products/ingenuity-pathway-analysis).

The top network (Network 1) in HCT116 had a network score (NS) of 124, and contains 124 focus molecules which include AURKA, AURKB, BBC3, CCNA2, CDK6, CDKN2B, E2F1, MCM2, MCM10, PLK1, POLA1, RAD51, RFC3, RRM2, TOP2A, TYMS (Fig. 6). Many of the focus molecules in this network were downregulated. Network 2 (*NS* = 73) in HCT116 has 119 focus molecules which include BIRF5, CCNB1, CDC25A, CDK2, CDKN1A, DDIT3, GADD45A, HMOX1, JUN, NDRG1, PCNA, while network 4 (*NS* = 66) has 84 focus molecules which include AURKB, CCNA2, CDKN1A, GINS1, KIF11, MXD1, PLK1, POLE2, TP53. Although NUPR1 was not considered as a focus molecule in network 4 (due to its MA_FC value of +1.27), IPA network analysis nevertheless indicated NUPR1 interacted with many other focus molecules in the network, and most of them were downregulated (Fig. 7), an observation similar to that seen in HepG2 (Fig. 8) (see below).

In HepG2, the top network (*NS* = 122) has 122 focus molecules which include AURKA, BUB1, CCNA2, CDK6, CDKN2B, CHEK1, GINS2, GMNN, MCM3, MCM10, NUPR1, POLA2, RAD51, RFC3, RRM2, SKP2, TOP2A (Fig. 8). The network figure shows NUPR1 (MA_FC +2.40) interacted with many other focus molecules, and many of them were downregulated. Identification of NUPR1 as a focus molecule here is supportive of the earlier finding, i.e., upstream regulator analysis, where NUPR1 was predicted by IPA to be the top upstream regulator in both HepG2 and HCT116 cells. Network 2 (*NS* = 110) in HepG2 has 116 focus molecules which include BBC3, DDIT4, ERN1, GABARAPL1, NDRG1, while network 3 (*NS* = 68) has 90 focus molecules which include BAK1, CCNB1, CDK1, CDKN1A, DDIT3, DUSP1, GADD45B, HMOX1, JUN, PCNA.

## Discussion

In the present study, the anticancer activity of MP-HX against HepG2 and HCT116 cells was characterised through microarray gene expression profiling followed by bioinformatics data analysis using TAC and IPA software. The microarray data was validated by performing RT-qPCR assay on selected genes that were differentially regulated. The direction of gene expression changes for the 17 genes assayed through RT-qPCR was found to be consistent with the microarray data.

TAC software analysis revealed that MP-HX significantly regulated many Wikipathways (WP) associated with cell cycle, cell proliferation and cell death. The pathways that were modulated include retinoblastoma in cancer (RIC), G_1_ to S cell cycle control (G_1_SCC), cell cycle (CC), DNA damage response (DDR) and apoptosis (AP).

The “Retinoblastoma in cancer Wikipathway” (RIC-WP) was the top pathway that was significantly regulated in both cell lines (Figs. S2 and S3). The RIC-WP mainly consists of Rb family protein pRb (also known as RB1), cyclin dependent kinases (CDKs), cyclin dependent kinase inhibitors and E2F proteins. The pRb protein acts as a tumor suppressor by inhibiting the function of E2F transcription factors, leading to inhibition of cell cycle progression (Giacinti & Giordano, 2006). The presence of CDKs in the G_1_ phase of the cell cycle inactivates pRb through phosphorylation, allowing the cells to enter S-phase and initiate DNA replication (Henley & Dick, 2012). The phosphorylation of pRb by CDKs are prevented by CDK inhibitors INK4 (p15, p16, p18, and p19) and Cip/Kip (p21, p27 and p57) (Asghar et al., 2015). In both cell lines, the concomitant decrease in the expression of E2F, CDK1, CDK2, CDK6, CCNA2, CCNB1, CCNB2, CCNE1 and CCNE2 as observed in the dataset may have conferred a synergistic effect, leading to MP-HX antiproliferative activity.

CDKN1A (p21) is an inhibitor of CDK in RIC-WP. CDKN1A expression was upregulated in both HCT116 (qPCR_FC +4.44) and HepG2 (qPCR_FC +2.03). CDKN1A is a sensor and effector of multiple anti-proliferative signals and capable of inducing cell cycle arrest (Abbas & Dutta, 2009). MP-HX also induced downregulation of E2F and CDKs expression in both HepG2 and HCT116 (Figs. S2 and S3). These facts are in accordance with CDKN1A upregulation as noted above, and these events are likely to contribute to the inhibition of G_1_/S transition, leading to anti-proliferative effect by MP-HX in both cell lines.

Cyclin A2 (CCNA2) is a component of RIC-WP. CCNA2 was downregulated by MP-HX in both HCT116 (qPCR_FC −18.89) and HepG2 (qPCR_FC −6.18) (Figs. S2 and S3). This gene is widely expressed in many tissues with important roles in mediating G_1_-S and G_2_/M transitions (Hochegger, Takeda & Hunt, 2008). Cyclin A2 promotes DNA replication during S-phase by activating CDK2. It also activates CDK1 to induce mitotic entry (Wolgemuth, 2008; Hochegger, Takeda & Hunt, 2008). CCNA2 overexpression has been reported in numerous types of cancers (Uhlen et al., 2010).

Ribonucleotide Reductase Regulatory Subunit-M2 (RRM2) is another component of RIC-WP that was downregulated by MP-HX in both HCT116 (qPCR_FC −7.45) and HepG2 (qPCR_FC −6.18). RRM2 is a subunit of ribonucleotide reductase (RNR). It catalyzes conversion of NDP to dNDP, which is pivotal for the supply of nucleotides for DNA synthesis and cell proliferation. RRM2 can be classified as an oncogene as its overexpression in transgenic mice promoted tumour development (Furuta et al., 2010). RRM2 overexpression in epithelial ovarian carcinoma (EOC) cases predicts a shorter overall survival. Knockdown of RRM2 in EOC cell lines inhibited its proliferation and triggers cellular senescence (Aird et al., 2014).

MP-HX also modulated “G_1_ to S cell cycle control” Wikipathway (G_1_SCC-WP) in both cell lines (Figs. S4 and S5). Cyclin Dependent Kinase Inhibitor 2B (CDKN2B) is a component of G_1_SCC-WP that was upregulated after treatment with MP-HX in HCT116 (qPCR_FC +9.82) and HepG2 (qPCR_FC +3.52). CDKN2B encodes a cyclin-dependent kinase inhibitor, which can form a complex with CDK4 or CDK6, and prevents the activation of the CDK kinases (Santo, Siu & Raje, 2015). CDKN2B is capable of inhibiting CDK4/6 activity by preventing them from hyperphosphorylating RB proteins, leading to G_1_ cell cycle arrest (Kim & Sharpless, 2006). CDKN2B was reported to be deleted in a wide variety of human tumors (Kim & Sharpless, 2006). Inactivation of this gene promoted carcinogenesis in hepatocellular and colorectal carcinoma (Ren et al., 2015; Ishiguro et al., 2006).

GADD45A is another component gene in G_1_SCC-WP that was upregulated by MP-HX in HCT116 (qPCR_FC +5.49) and HepG2 (qPCR_FC +2.12). This gene is a member of growth arrest and DNA damage family of proteins (GADD). GADD45A upregulation is often induced by DNA damage and other stress signals associated with growth arrest and apoptosis (Salvador, Brown-Clay & Fornace Jr, 2013). GADD45A interactions with CDK1, cyclin B1 and p21 can inhibit cell cycle progression and induce apoptotic cell death through JNK/p38 pathway (Zhan, 2005). During G_2_/M checkpoint, GADD45A can arrest cell cycle progression by inhibiting cyclin-B1 and CDC2 interaction, preventing entry into M-phase. Repression of GADD45A expression alleviated G_2_/M arrest (Rosemary Siafakas & Richardson, 2009).

The “Cell cycle Wikipathway” (CC-WP) was also significantly modulated by MP-HX treatment in both cell lines (Figs. S6 and S7). The CC-WP member genes include cyclins and CDKs proteins, which are responsible for the promotion of cell cycle progression. These proteins are frequently overexpressed in numerous types of cancer (Asghar et al., 2015). In both cell lines, MP-HX downregulated many of the cyclins and CDKs genes, including CCNA2, CCNB1, CCNB2, CCNE2, CDK1, 2 and 6. Downregulation of these genes likely contributed to MP-HX inhibition of cell cycle progression in the cancer cells. Many anticancer drugs have been reported to exert anticancer effect by arresting cell cycle progression at G_1_/S and G_2_/M transition points, and this is achieved through the inhibition of key proteins that promote cell cycle progression (Santo, Siu & Raje, 2015; Dominguez-Brauer et al., 2015).

The CC-WP also include minichromosome maintenance (MCM), S-phase kinase-associated protein 2 (SKP2) and Polo-like kinase 1 (PLK1) genes. These genes were also downregulated by MP-HX in both cell lines. The MCM family of proteins play important roles in replication, transcription, checkpoint response and cancer progression (Forsburg, 2004; Deegan & Diffley, 2016). MCM2 was markedly downregulated by MP-HX treatment in HCT116 (qPCR_FC −32.97) and HepG2 (qPCR_FC −12.90). MCM2 is a component member of Mcm2-7 complex. The complex unwinds parental DNA to form single-stranded DNA template during replication (Simon & Schwacha, 2014). MCM2 overexpression has been reported in colorectal, glioma and oral squamous cell carcinomas (Guzińska-Ustymowicz et al., 2009; Razavi et al., 2015; Hua et al., 2014). MCM10 was also significantly downregulated by MP-HX treatment in HCT116 (qPCR_FC −23.99) and HepG2 (qPCR_FC −21.03). MCM10 is thought to be essential for activating MCM2-7 helicase activity, since the unwinding of origins of replication is defective in the absence of MCM10 (Deegan & Diffley, 2016). MCM10 and MCM2 overexpression have been reported in urothelial and cervical cancers (Li et al., 2016a; Das et al., 2013). Of note, MP-HX also downregulated other MCMs, which include MCM3 (MA_FC −4.15, −2.90) and MCM7 (MA_FC −2.79, −1.53) in HCT116 and HepG2, respectively.

SKP2 is a component of ubiquitin–proteasome system (UPS), which plays vital roles in regulating the degradation of various cellular proteins (Wang et al., 2012). This gene was downregulated in both HCT116 (qPCR_FC −10.90) and HepG2 (qPCR_FC -28.53). SKP2 is a subunit in Skp1–Cullin1–F-box (SCF) E3 ligase complex. Skp2 is an F-box protein of the SCF complex SCF^skp2^ E3 ligase, which targets many tumor suppressor proteins (eg. p27, p21, p57, TOB1, FOXO1) for degradation. For this reason, it is not surprising that overexpression of SKP2 is observed in diverse type of human cancers (Wang et al., 2012).

Polo-like kinase 1 (PLK1) is a member of polo-like kinase family. PLK1 was also downregulated in HCT116 (qPCR_FC -19.52) and HepG2 (qPCR_FC -14.94). PLK1 roles include regulation of mitotic entry, centrosome separation and maturation, kinetochore attachment and cytokinesis (Schmucker & Sumara, 2014). PLK1 was reported to be overexpressed in various cancers and thought to confer tolerance of cells to cancer associated cellular stress (Strebhardt, 2010). Inhibition of PLK1 was also reported to induce anticancer effect in hepatocellular carcinoma and retinoblastoma cell lines (Xu et al., 2017; Schwermer et al., 2017). Up to this point, it is interesting to note that in both cell lines, MP-HX downregulated all six top-ranked genes (PLK1, MCM2, MCM3, MCM7, MCM10 and SKP2) that were proposed to be cancer-associated (Wu, Zhu & Zhang, 2012).

MP-HX also modulated many components genes of “DNA damage response Wikipathway” (DDR-WP) in both cell lines. The expression of genes in DDR-WP can be induced by genotoxic agents or environmental stress such as UV and ionizing radiation, and the response could lead to induction of cell cycle arrest and mobilisation of DNA repair mechanisms to circumvent DNA damage (O’Connor, 2015). In both HCT116 and HepG2, MP-HX downregulated some of the genes in DDR-WP, which include CHEK1, RAD51, CDC25A, cyclins and CDKs, while CDKN1A, BBC3 and GADD45A expression were upregulated. With regards to their functions, CHEK1, RAD51 and CDC25A have been suggested to promote tumor development, while GADD45A can act as a tumor suppressor (Hosoya & Miyagawa, 2014; Broustas & Lieberman, 2014). CDKN1A, as discussed earlier, can induce cell cycle arrest through inhibition of cyclins and CDKs. BBC3 is a pro-apoptotic protein which is capable of suppressing cancer development (Yu & Zhang, 2008), and a more detailed description of its role is discussed below.

MP-HX treatment also modulated “Apoptosis Wikipathway” (AP-WP) in both cell lines. Apoptosis is a form of programmed cell death, characterized by chromatin condensation, cytoplasmic shrinkage, membrane blebbing and nuclear fragmentation (Wong, 2011). Many type of cancers are known to evade apoptosis for their survival (Hanahan & Weinberg, 2011). Apoptotic signaling is modulated by BCL-2 anti-apoptotic and pro-apoptotic family proteins, inhibitor of apoptosis proteins (IAP), p53 tumor suppressor, caspases and death receptor signaling (Wong, 2011). The Bcl-2 family of proteins is one of the major regulators of apoptosis, namely the intrinsic pathway of apoptosis. This family contains pro-apoptotic and anti-apoptotic (or pro-survival) protein members (Delbridge & Strasser, 2015). An imbalance in the amount of BCL-2 family of proteins, overexpression of IAP proteins, inhibition of caspases, reduced p53 function and impaired death receptor signaling are responsible for the development of cancer and chemoresistance (Pistritto et al., 2016). Therefore, targeting proteins which modulate apoptosis is of key interest in cancer therapy (Goldar et al., 2015). Apoptosis modulating drugs may inhibit BCL-2 anti-apoptotic proteins, IAP proteins or activate BCL-2 pro-apoptotic proteins, caspase enzymes and p53 functions to confer anticancer effect (Wong, 2011).

MP-HX treatment induced upregulation of BBC3 in HCT116 (qPCR_FC +4.48) and HepG2 (qPCR_FC +2.68). BBC3 is a pro-apoptotic gene of the BCL-2 family. At the same time, MP-HX treatment induced downregulation of BIRC5, which is an inhibitor of apoptosis (IAP) protein, in HCT116 (MA_FC −3.44) and HepG2 (MA_FC −1.82). BBC3 is known to facilitate p53-dependent or independent apoptosis (Yu & Zhang, 2008) while BIRC5 was reported to be overexpressed in various type of cancers (Kelly et al., 2011). BBC3 antagonizes anti-apoptotic members of Bcl-2 to promote cell death. BBC3 colocalizes in mitochondrial membrane to inhibit anti-apoptotic Bcl-2 family members, while activating pro-apoptotic Bax/Bak, causing outer mitochondrial membrane permeabilization and leading the initiation of mitochondrial pathway of apoptosis. Inhibition or deletion of BBC3 impairs apoptosis and contributing to the development of cancer and chemo-resistance (Hikisz & Kilianska, 2012).

MP-HX also induced the upregulation of another AP-WP member, PMAIP1 (NOXA) in HCT116 (MA_FC +1.69) and HepG2 (MA_FC +3.10). In HepG2 cells, MP-HX upregulated pro-apoptotic gene BAK1 (MA_FC +2.14), another member of AP-WP. NOXA is capable of inactivating anti-apoptotic members of BCL-2 family, allowing BAK and BAX to interact with pro-apoptotic members of BCL-2 and leading to apoptosis (Hata, Engelman & Faber, 2015; Albert, Brinkmann & Kashkar, 2014).

Lymphoid specific helicase (HELLS) is another component member of AP-WP that was downregulated by MP-HX in both HCT116 (qPCR_FC −6.43) and HepG2 (qPCR_FC −3.01). HELLS demonstrated diverse functions, which include epigenetics regulation of development, co-activator of E2F to stimulate cell growth and DNA repair (Mjelle et al., 2015). HELLS can promote cancer, as it enhances growth, migration, and invasion of nasopharyngeal carcinoma cell lines (He et al., 2016). Overexpression of HELLS was also reported in prostate and oropharyngeal squamous cell carcinomas (Von Eyss et al., 2012); (Janus et al., 2011). Increased HELLS expression was associated with poor prognosis in patients with renal cell carcinoma (Chen et al., 2017).

JUN (cJUN) is another component member of AP-WP that was upregulated by MP-HX in HCT116 (qPCR_FC +9.45) and HepG2(qPCR_FC +6.00). JUN proto-oncogene is a component of the AP-1 transcription factor that demonstrated cancer promoting, as well as apoptotic induction activities (Eferl et al., 2003; Fan & Chambers, 2001; Dhanasekaran & Reddy, 2008; Bossy-Wetzel, Bakiri & Yaniv, 1997). In glioma cells treated with temozolomide and nimustine, apoptosis was achieved through c-JUN mediated activation of BIM (BCL-2 interacting mediator of cell death) (Tomicic et al., 2015). The anticancer activity of tylophorine, an alkaloid from *Tylophora indica* was also demonstrated to be mediated by c-JUN in HONE-1, NUGC-3 and HepG2 carcinoma cells. The upregulation of c-JUN by tylophorine resulted to cyclin A2 transcriptional repression, leading to G_1_ cell cycle arrest (Yang et al., 2013). The induction of pro-apoptotic and downregulation of anti-apoptotic genes suggests that MP-HX promoted network signaling or transcriptome profile favoring apoptosis induction. This finding is consistent with our previous report, which demonstrated apoptosis induction of MP-HX on four different cancer cell lines (Kabir et al., 2017).

IPA software analysis also revealed that MP-HX modulated many canonical pathways, biological functions and gene networks linked to cell cycle, cell proliferation, DNA replication, DNA damage, cell death and apoptosis in both HepG2 and HCT116 cell lines. IPA ranked nuclear protein 1 (NUPR1) as the top upstream regulator that was activated in both cell lines by MP-HX treatment. The microarray data also showed that MP-HX upregulated NUPR1 expression in both HCT116 (MA_FC +1.27) and HepG2 (MA_FC +2.40) cell lines. NUPR1 is a transcription factor that has been reported to show tumor inhibiting and promoting activities. NUPR1 was able to inhibit tumor progression in brain and prostate cancers, as well as being overexpressed in several other cancers (Goruppi & Iovanna, 2010). A recent study reported NUPR1 acted as a negative regulator of tumor repopulating cells (TRCs) growth (Jia et al., 2016). The above observations suggest NUPR1 had a significant role in mediating MP-HX anticancer activity. Future study could investigate this hypothesis by silencing NUPR1 expression in MP-HX treated cells.

IPA analysis also predicted tumor protein 53 (TP53) as another top upstream regulator that was activated in both cell lines. TP53 is a classical tumor suppressor gene which is frequently mutated in cancers (Leroy et al., 2014). TP53 and its target genes are renowned for their involvement in cell cycle arrest, DNA repair and apoptosis (Fischer, 2017). IPA also predicted RABL6 (RAB, member RAS oncogene family like 6) as another top upstream regulator, with activation *z*-scores that are suggestive of its inhibition in both cell lines. The microarray data showed that RABL6 was mildly downregulated in HepG2 and HCT116 (MA_FC of −1.11 and −1.28, respectively). RABL6 has been reported to promote proliferation of osteosarcoma and pancreatic neuroendocrine tumor through inhibition of retinoblastoma 1 (pRb) (Tang et al., 2016; Hagen et al., 2014). RABL6 also enhanced MDM2-induced TP53 degradation (Lui et al., 2013). These highlight RABL6 tumor promoting properties. The activation or inhibition of the above transcriptional regulators in both cell lines likely played significant roles in modulating the antiproliferative effect induced by MP-HX, and a more detailed investigation on their contributions could be explored in future studies.

IPA software network analysis suggested N-myc Downstream Regulated 1 (NDRG1) as a focus molecule in both HCT116 (network 2) and HepG2 (network 2) cells. NDRG1 expression was upregulated by MP-HX in both HCT116 (qPCR_FC +6.52) and HepG2 (qPCR_FC +5.69) cells. NDRG1 demonstrated anti-oncogenic function and was proposed to be a metastasis suppressor in diverse type of cancers (Fang et al., 2014). NDRG1 is capable of blocking metastasis by activating cell adhesion molecules (E-cadherin, β-catenin), and inhibiting snail/slug, Wnt signalling and ROCK1/pMLC2 pathways (Bae et al., 2013). GINS complex subunit 2 (GINS2) is another focus molecule highlighted by IPA network analysis. GINS2 is a component member of network 1 in HepG2 and network 5 in HCT116 cells, and its expression was downregulated in both HCT116 (qPCR_FC −6.73) and HepG2 (qPCR_FC −2.19) cell lines. GINS2 is a component of ‘GINS complex’, a tetrameric complex with crucial roles in the initiation of DNA replication, including correct assembly and maintenance of DNA replication forks and its progression during replication (Gambus et al., 2006). GINS2 upregulation was associated with poor prognosis and reduced survival in early-stage cervical cancer (Ouyang et al., 2017). In breast cancer patients, elevated GINS2 transcript level was associated with poor relapse-free and distant metastasis-free survival (Zheng et al., 2014).

The present study shows that MP-HX exert anticancer activity by inducing gene expression changes in HCT116 and HepG2 cells that can promote antiproliferative or anticancer effect. Phytochemical compounds with anticancer properties which have been identified in MP include β-sitosterol, lupeol, oleanolic acid, kokusaginine and genistein (Baskar et al., 2010; Rauth et al., 2016; Shaari et al., 2011; Tiwari, Brunton & CS, 2013; Li et al., 2016b; Molnar et al., 2013). Further studies are warranted to identify the exact constituent(s) that is/are responsible for MP anticancer activity.

## Conclusions

The present study showed that MP-HX conferred its anticancer activity by modulating the expression of various key genes regulating DNA replication, cell cycle progression, cell growth/proliferation and programmed cell death in a direction favoring anticancer effect. The transcriptome profiles induced by MP-HX include upregulation of pro-apoptotic, cell cycle arresting and metastasis suppression genes and downregulation of genes that promote anti-apoptotic effect, cell cycle progression, tumor development and progression. The ability of MP-HX to downregulate all six top-ranked genes that were proposed to be cancer-associated is also supportive of its anticancer activity (Wu, Zhu & Zhang, 2012). The findings in the present study provide novel insights on the anticancer activity of MP and further project its potential use as a nutraceutical agent for cancer therapeutics.

##  Supplemental Information

10.7717/peerj.5203/supp-1 Data S1 Wikipathways (WPs) that were significantly regulated (*p* < 0.05) in HCT116 and HepG2 cells after MP-HX treatment

10.7717/peerj.5203/supp-2 Data S2 IPA software analyses - networks enriched in HCT116 and HepG2, induced by MP-HX treatment

10.7717/peerj.5203/supp-3 Figure S1 Validation of microarray data through RT-qPCR The bar chart shows gene expression fold changes (FC) obtained from microarray and real-time PCR assays. The data for each assay was derived from three biological replicates. For each qPCR assay, the data was normalized to RPS29 expression and the value represent mean qPCR_FC ±SD (*n* = 3).

10.7717/peerj.5203/supp-4 Figure S2 Modulation of ‘Retinoblastoma In Cancer’ Wikipathway (RIC-WP) component genes by MP-HX in HCT116 cells. The figure shows component genes of RIC-WP that were differentially regulated by MP-HX in HCT116 cells. The genes in the pathway are colored red (MA_FC ≥ + 2.0), or green (MA_FC ≥ − 2.0), or depicted as grey hashed boxes (MA_FC < ±2).

10.7717/peerj.5203/supp-5 Figure S3 Modulation of ‘Retinoblastoma In Cancer’ Wikipathway (RIC-WP) component genes by MP-HX in HepG2 cells The figure shows component genes of RIC-WP that were differentially regulated by MP-HX in HepG2 cells. The genes in the pathway are colored red (MA_FC ≥ + 2.0), or green (MA_FC ≥ − 2.0), or depicted as grey hashed boxes (MA_FC < ±2).

10.7717/peerj.5203/supp-6 Figure S4 Modulation of ‘G_1_ to S Cell Cycle Control’ Wikipathway (G_1_SCC-WP) component genes by MP-HX inHCT116 cells The figure shows component genes in G_1_SCC-WP that were differentially regulated by MP-HX treatment in HCT116 cells. The genes in the pathway are colored red (MA_FC ≥ + 2.0), or green (MA_FC ≥ − 2.0), or depicted as grey hashed boxes (MA_FC < ±2).

10.7717/peerj.5203/supp-7 Figure S5 Modulation of ‘G_1_ to S Cell Cycle Control’ Wikipathway(G_1_SCC-WP) component genes by MP-HX inHepG2 cells. The figure shows component genes of G_1_SCC-WP that were differentially regulated by MP-HX in HepG2 cells.The genes in the pathway are colored red (MA_FC ≥ + 2.0), or green (MA_FC ≥ − 2.0), or depicted as grey hashed boxes (MA_FC < ±2).

10.7717/peerj.5203/supp-8 Figure S6 Modulation of ‘Cell Cycle’ Wikipathway (CC-WP) component genes by MP-HX in HCT116 cells The figure shows component genes of CC-WP that were differentially regulated by MP-HX in HCT116 cells. The genes in the pathway are colored red (MA_FC ≥ + 2.0), or green (MA_FC ≥ − 2.0), or depicted as grey hashed boxes (MA_FC < ±2).

10.7717/peerj.5203/supp-9 Figure S7 Modulation of ‘Cell cycle’ Wikipathway (CC-WP) component genes by MP-HX in HepG2 cells The figure shows component genes of CC-WP that were differentially regulated by MP-HX in HepG2 cells. The genes in the pathway are colored red (MA_FC ≥ + 2.0), or green (MA_FC ≥ − 2.0), or depicted as grey hashed boxes (MA_FC < ±2).

10.7717/peerj.5203/supp-10 Figure S8 Modulation of ‘DNA Damage Response’ Wikipathway (DDR-WP) component genes by MP-HX in HCT116 cells The figure shows component genes in DDR-WP that were differentially regulated by MP-HX in HCT116 cells. The genes in the pathway are colored red (MA_FC ≥ + 2.0), or green (MA_FC ≥ − 2.0), or depicted as grey hashed boxes (MA_FC < ±2).

10.7717/peerj.5203/supp-11 Figure S9 Modulation of ‘DNA Damage Response’ Wikipathway (DDR-WP) component genes by MP-HX in HepG2 cells The figure shows component genes in DDR-WP that were differentially regulated by MP-HX in HepG2 cells. The genes in the pathway are colored red (MA_FC ≥ + 2.0), or green (MA_FC ≥ − 2.0), or depicted as grey hashed boxes (MA_FC < ±2).

10.7717/peerj.5203/supp-12 Figure S10 Modulation of ‘Apoptosis’ Wikipathway (AP-WP) component genes by MP-HX in HCT116 cells The figure shows component genes in AP-WP that were differentially regulated by MP-HX in HCT116 cells. The genes in the pathway are colored red (MA_FC ≥ + 2.0), or green (MA_FC ≥ − 2.0), or depicted as grey hashed boxes (MA_FC < ±2).

10.7717/peerj.5203/supp-13 Figure S11 Modulation of ‘Apoptosis’ Wikipathway (AP-WP) component genes by MP-HX in HepG2 cells The figure shows component genes in AP-WP that were differentially regulated (MA_FC ≥ ±2.00) by MP-HX in HepG2 cells. The genes in the pathway are colored red (MA_FC ≥ + 2.0), or green (MA_FC ≥ − 2.0), or grey (MA_FC < ±2).

10.7717/peerj.5203/supp-14 Figure S12 Modulation of diseases and biological functions by MP-HX in HCT116 cells The figure shows top category of diseases and biological functions that were modulated by MP-HX (FC ≥ ±2.0) in HCT116 cells, and they were ranked by IPA software based on -log (*p*-value) ≥5.0.

10.7717/peerj.5203/supp-15 Figure S13 Modulation of diseases and biological functions by MP-HX in HepG2 cells The figure shows top category of diseases and biological functions that were modulated by MP-HX (FC ≥ ±2.0) in HepG2 cells and they were ranked by IPA software based on -log (*p*-value) ≥5.0.

## Additional Information and Declarations

### Data Availability

The following information was supplied regarding data availability: Raw data has been uploaded into Gene Expression Omnibus (GEO) repository with the accession number given as GSE114743.
