# Supplementary figures and images for: Microarray gene expression profiling in colorectal (HCT116) and hepatocellular (HepG2) carcinoma cell lines treated with Melicope ptelefolia leaf extract reveals transcriptome profiles exhibiting anticancer activity
Source: PeerJ. 2018 Jul 18;6:e5203. doi: 10.7717/peerj.5203 (PMC6054789; doi:10.7717/peerj.5203)

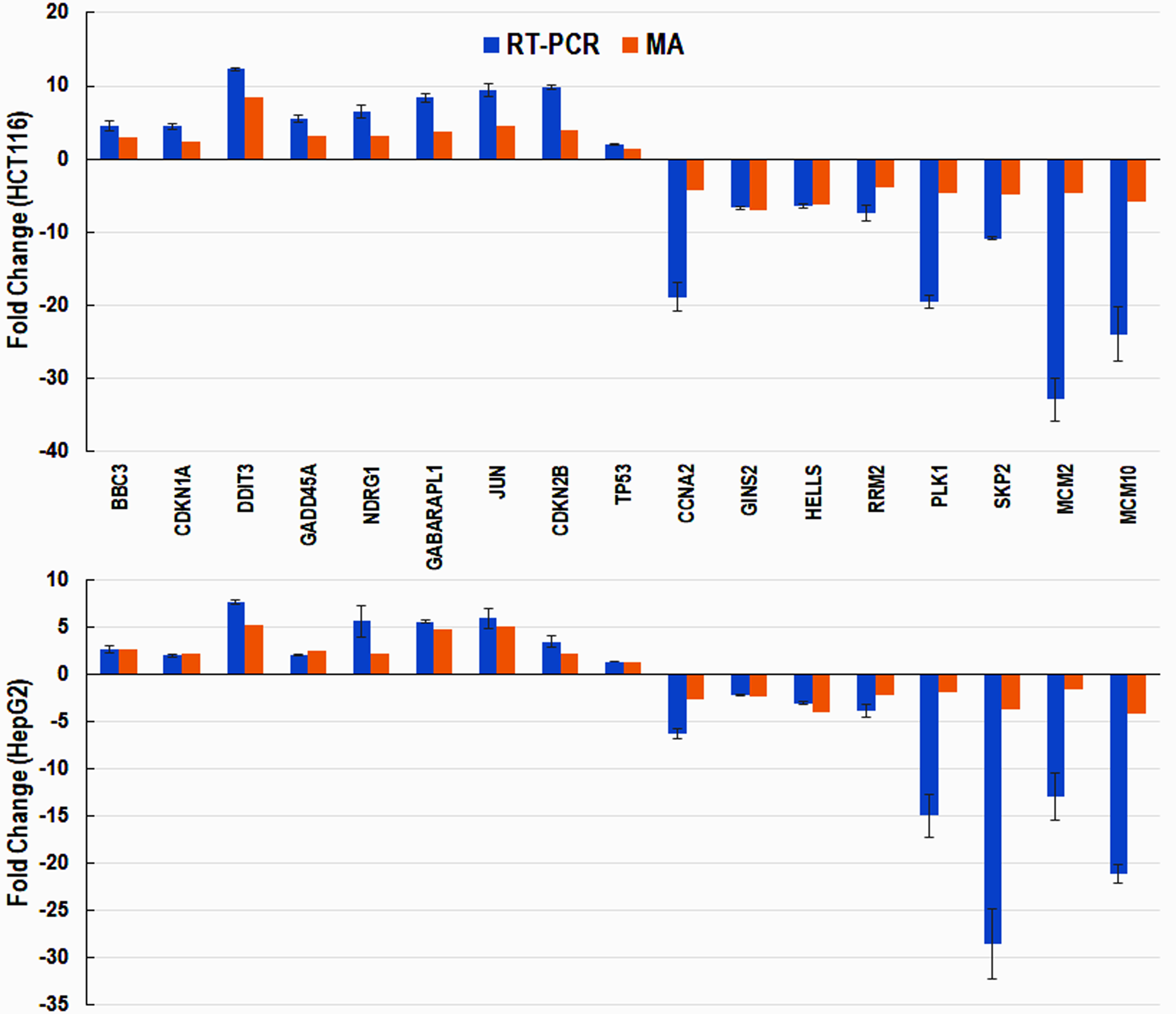

Supplement: Figure S1 — The bar chart shows gene expression fold changes (FC) obtained from microarray and real-time PCR assays. The data for each assay was derived from three biological replicates. For each qPCR assay, the data was normalized to RPS29 expression and the value represent mean qPCR_FC ±SD (n = 3). [file peerj-06-5203-s003.png]

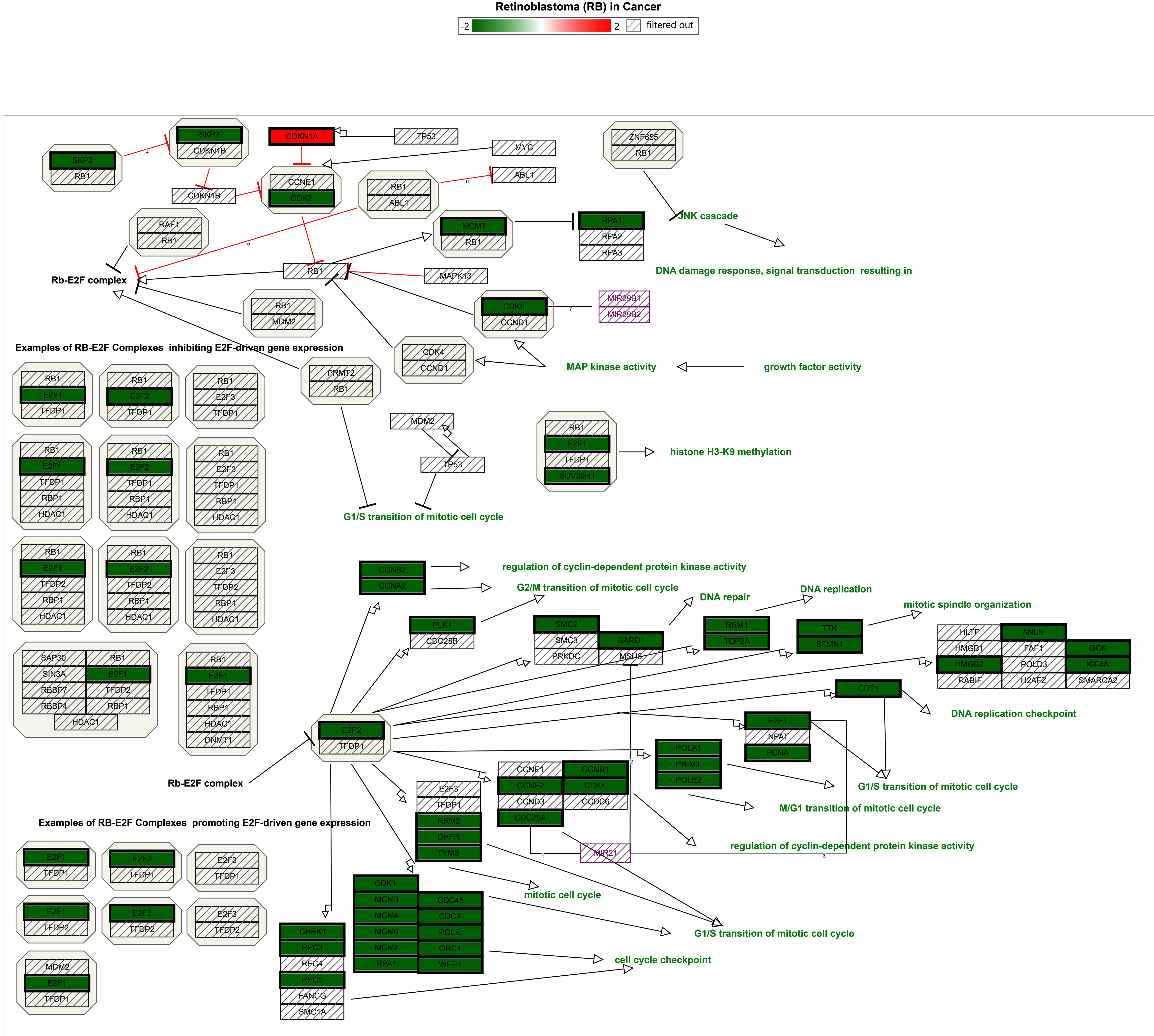

Supplement: Figure S2 — The figure shows component genes of RIC-WP that were differentially regulated by MP-HX in HCT116 cells. The genes in the pathway are colored red (MA_FC ≥ + 2.0), or green (MA_FC ≥ − 2.0), or depicted as grey hashed boxes (MA_FC < ±2). [file peerj-06-5203-s004.jpg]

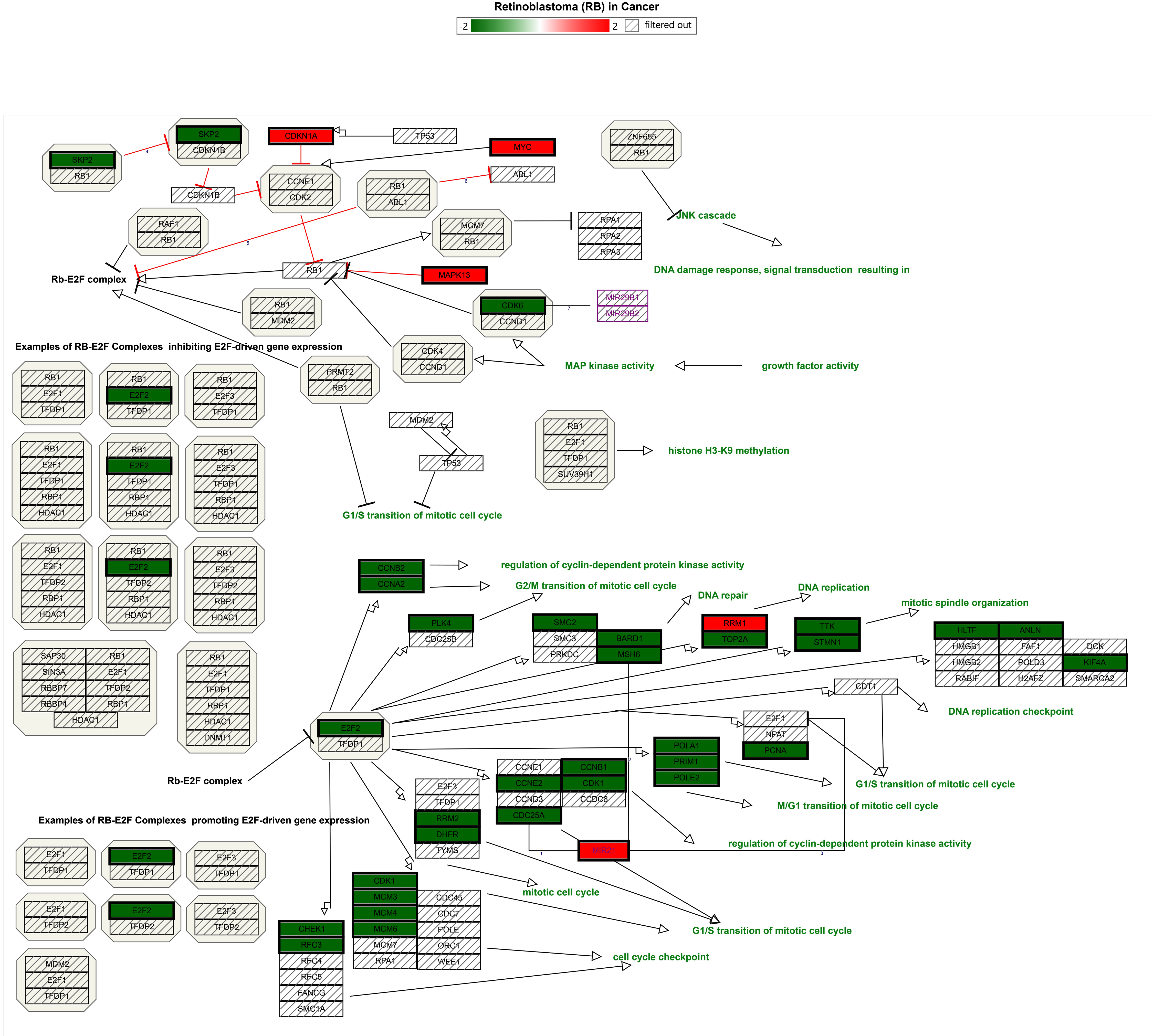

Supplement: Figure S3 — The figure shows component genes of RIC-WP that were differentially regulated by MP-HX in HepG2 cells. The genes in the pathway are colored red (MA_FC ≥ + 2.0), or green (MA_FC ≥ − 2.0), or depicted as grey hashed boxes (MA_FC < ±2). [file peerj-06-5203-s005.jpg]

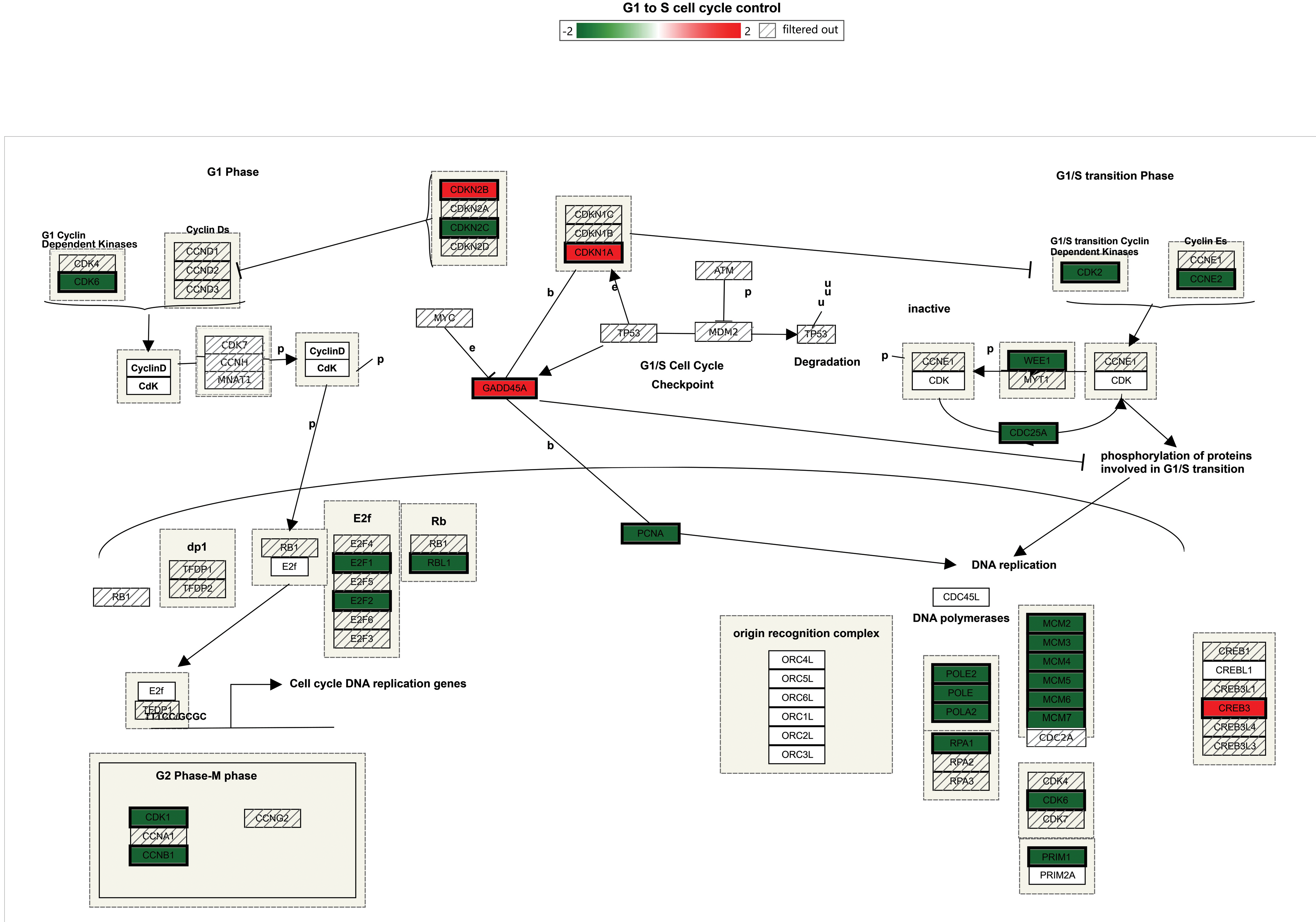

Supplement: Figure S4 — The figure shows component genes in G1SCC-WP that were differentially regulated by MP-HX treatment in HCT116 cells. The genes in the pathway are colored red (MA_FC ≥ + 2.0), or green (MA_FC ≥ − 2.0), or depicted as grey hashed boxes (MA_FC < ±2). [file peerj-06-5203-s006.png]

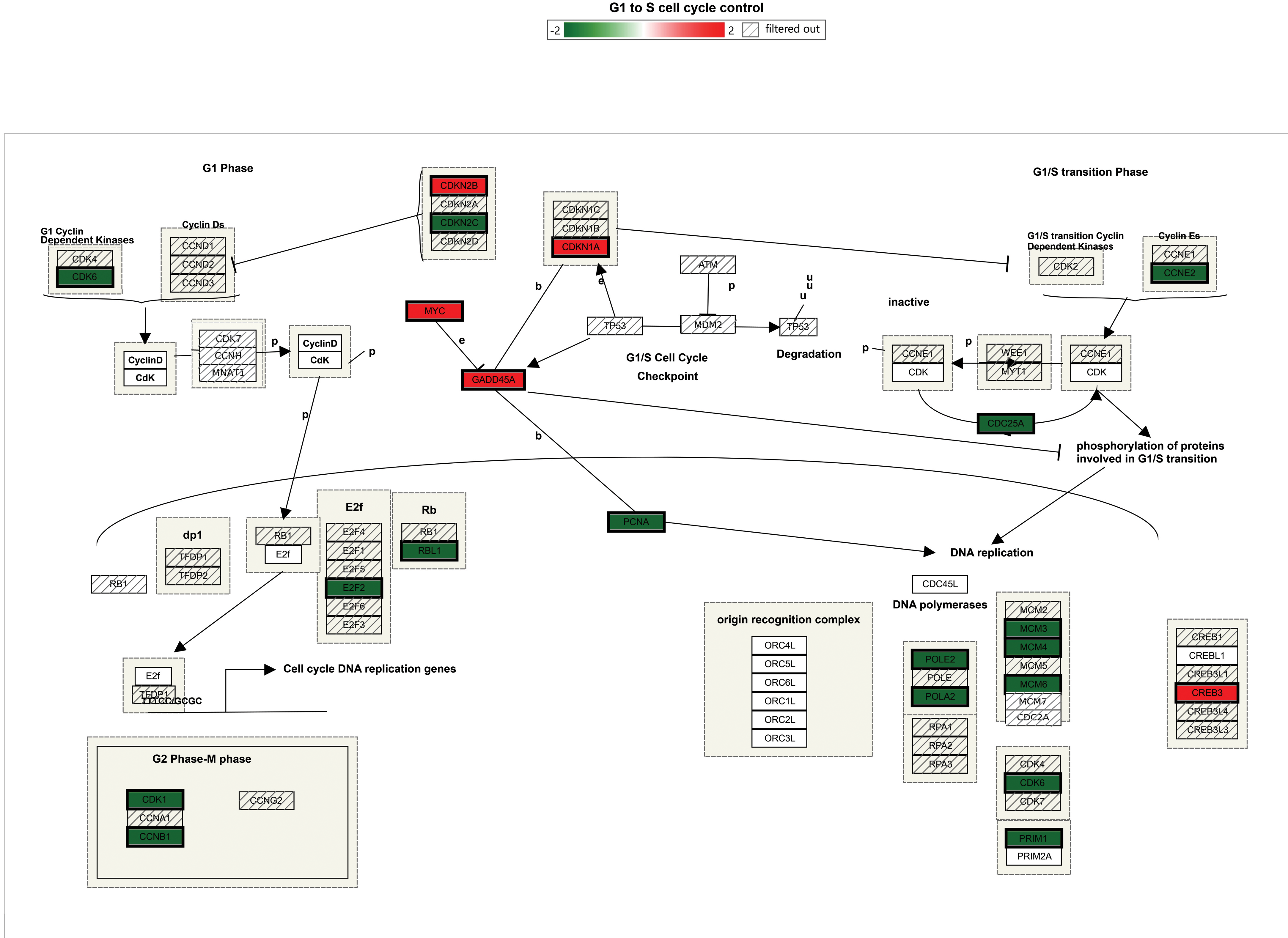

Supplement: Figure S5 — The figure shows component genes of G1SCC-WP that were differentially regulated by MP-HX in HepG2 cells.The genes in the pathway are colored red (MA_FC ≥ + 2.0), or green (MA_FC ≥ − 2.0), or depicted as grey hashed boxes (MA_FC < ±2). [file peerj-06-5203-s007.png]

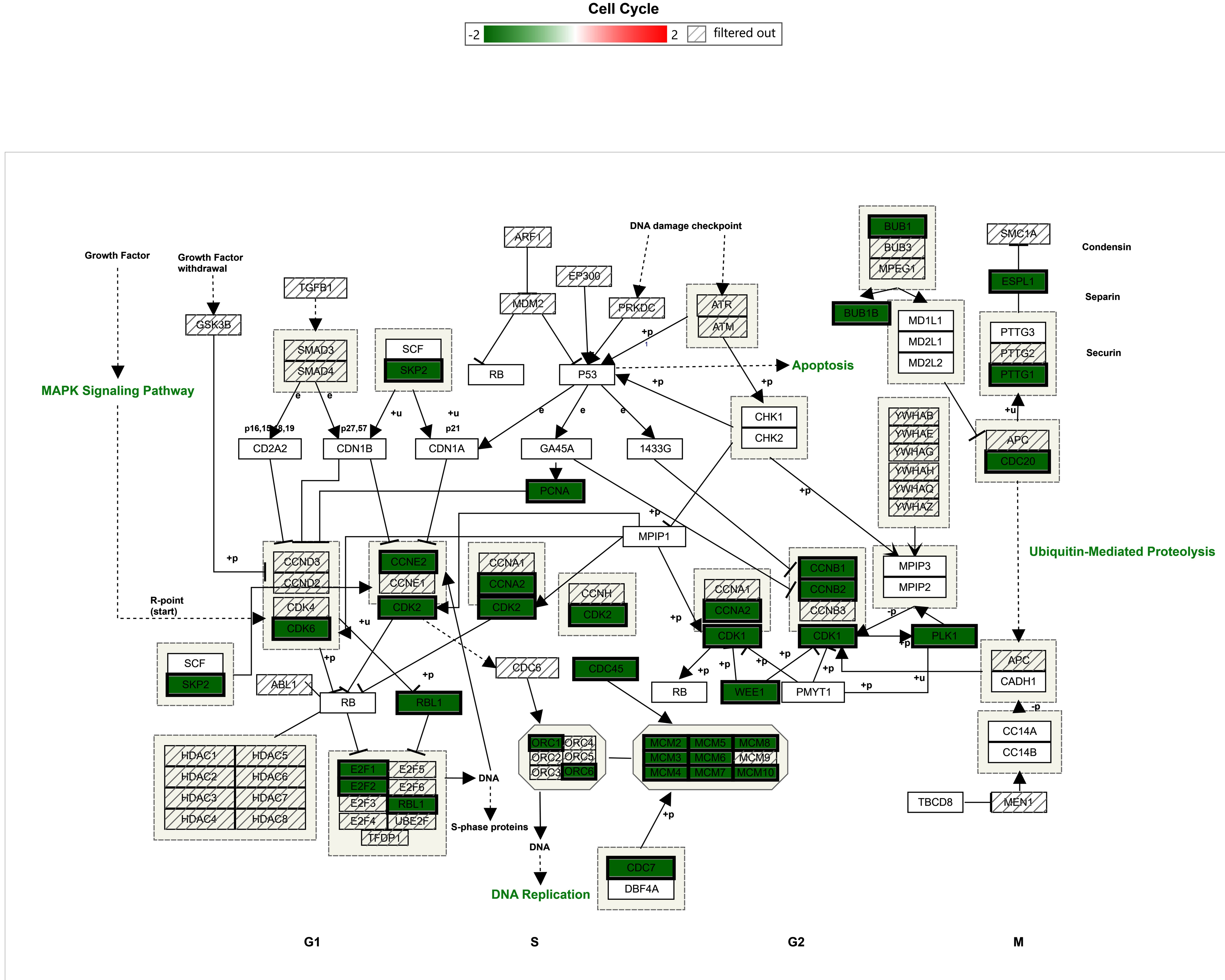

Supplement: Figure S6 — The figure shows component genes of CC-WP that were differentially regulated by MP-HX in HCT116 cells. The genes in the pathway are colored red (MA_FC ≥ + 2.0), or green (MA_FC ≥ − 2.0), or depicted as grey hashed boxes (MA_FC < ±2). [file peerj-06-5203-s008.jpg]

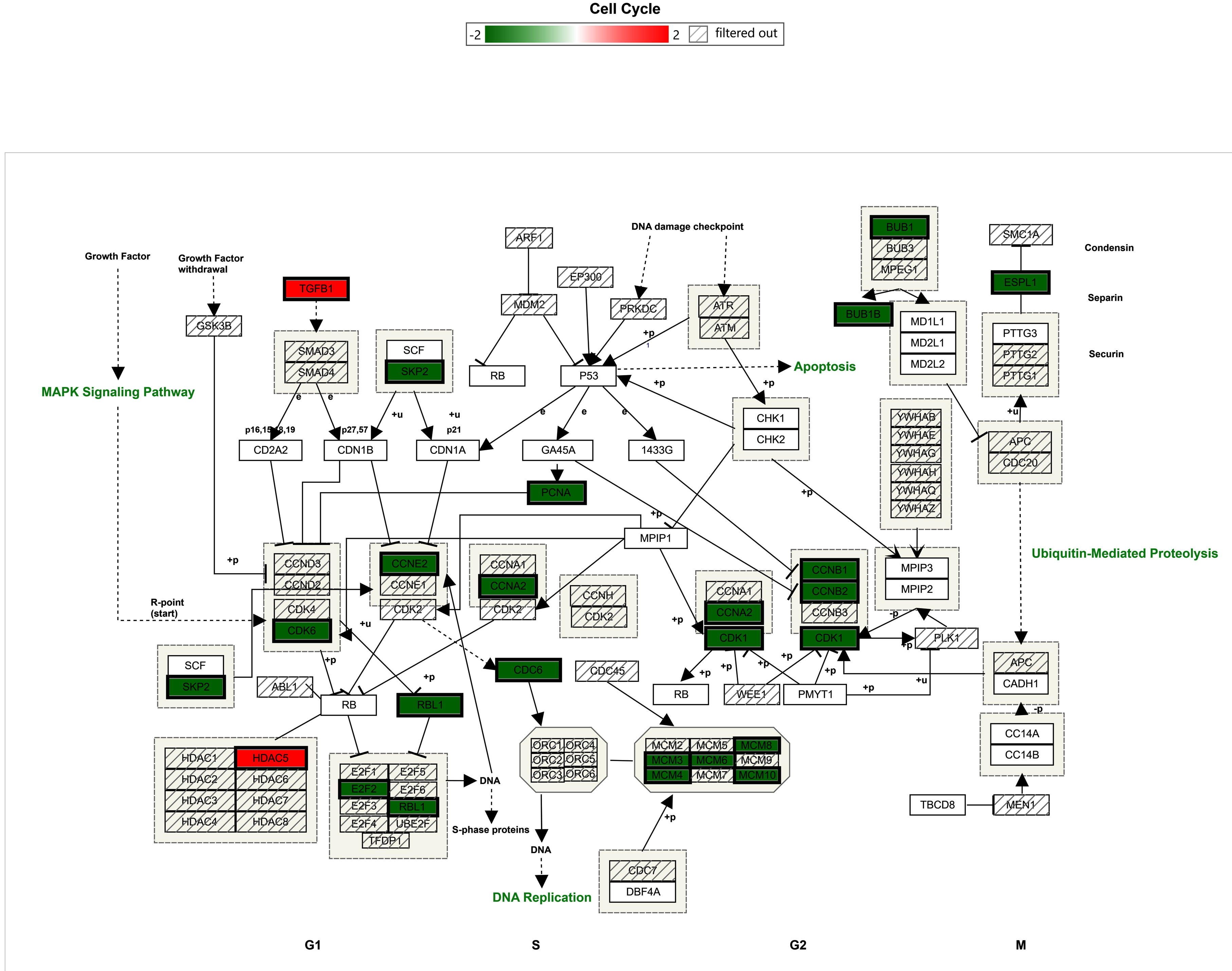

Supplement: Figure S7 — The figure shows component genes of CC-WP that were differentially regulated by MP-HX in HepG2 cells. The genes in the pathway are colored red (MA_FC ≥ + 2.0), or green (MA_FC ≥ − 2.0), or depicted as grey hashed boxes (MA_FC < ±2). [file peerj-06-5203-s009.jpg]

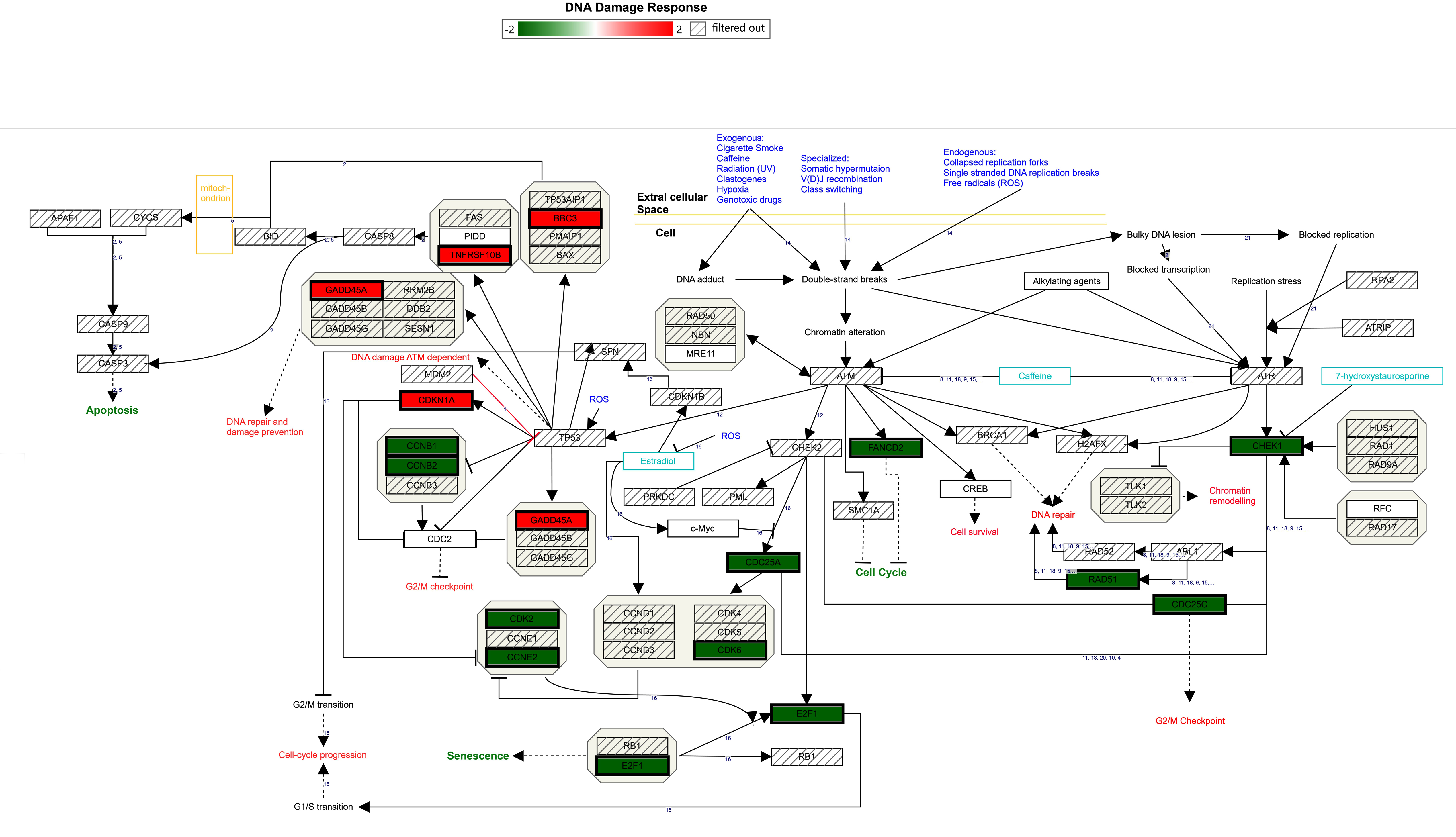

Supplement: Figure S8 — The figure shows component genes in DDR-WP that were differentially regulated by MP-HX in HCT116 cells. The genes in the pathway are colored red (MA_FC ≥ + 2.0), or green (MA_FC ≥ − 2.0), or depicted as grey hashed boxes (MA_FC < ±2). [file peerj-06-5203-s010.jpg]

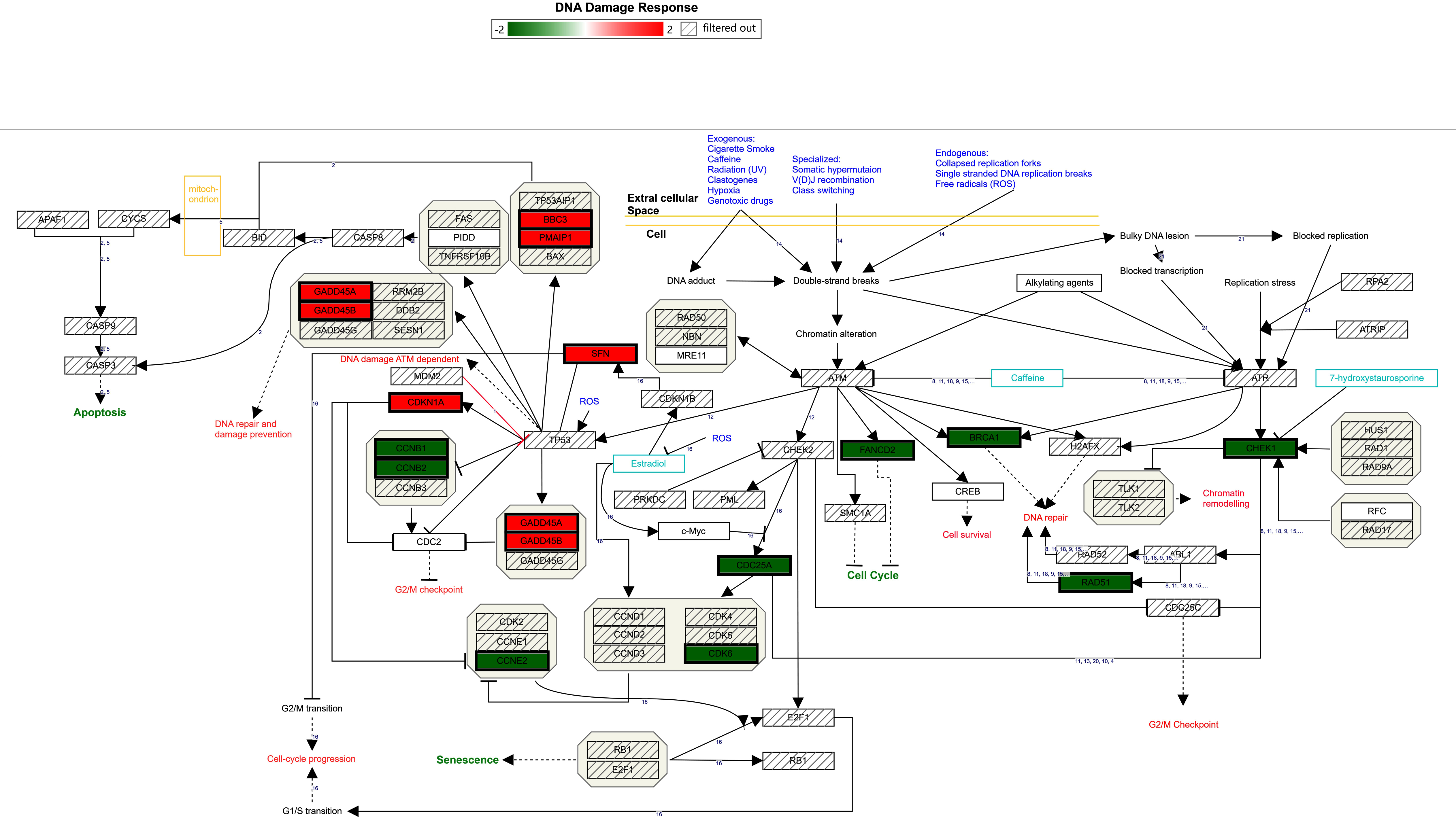

Supplement: Figure S9 — The figure shows component genes in DDR-WP that were differentially regulated by MP-HX in HepG2 cells. The genes in the pathway are colored red (MA_FC ≥ + 2.0), or green (MA_FC ≥ − 2.0), or depicted as grey hashed boxes (MA_FC < ±2). [file peerj-06-5203-s011.jpg]

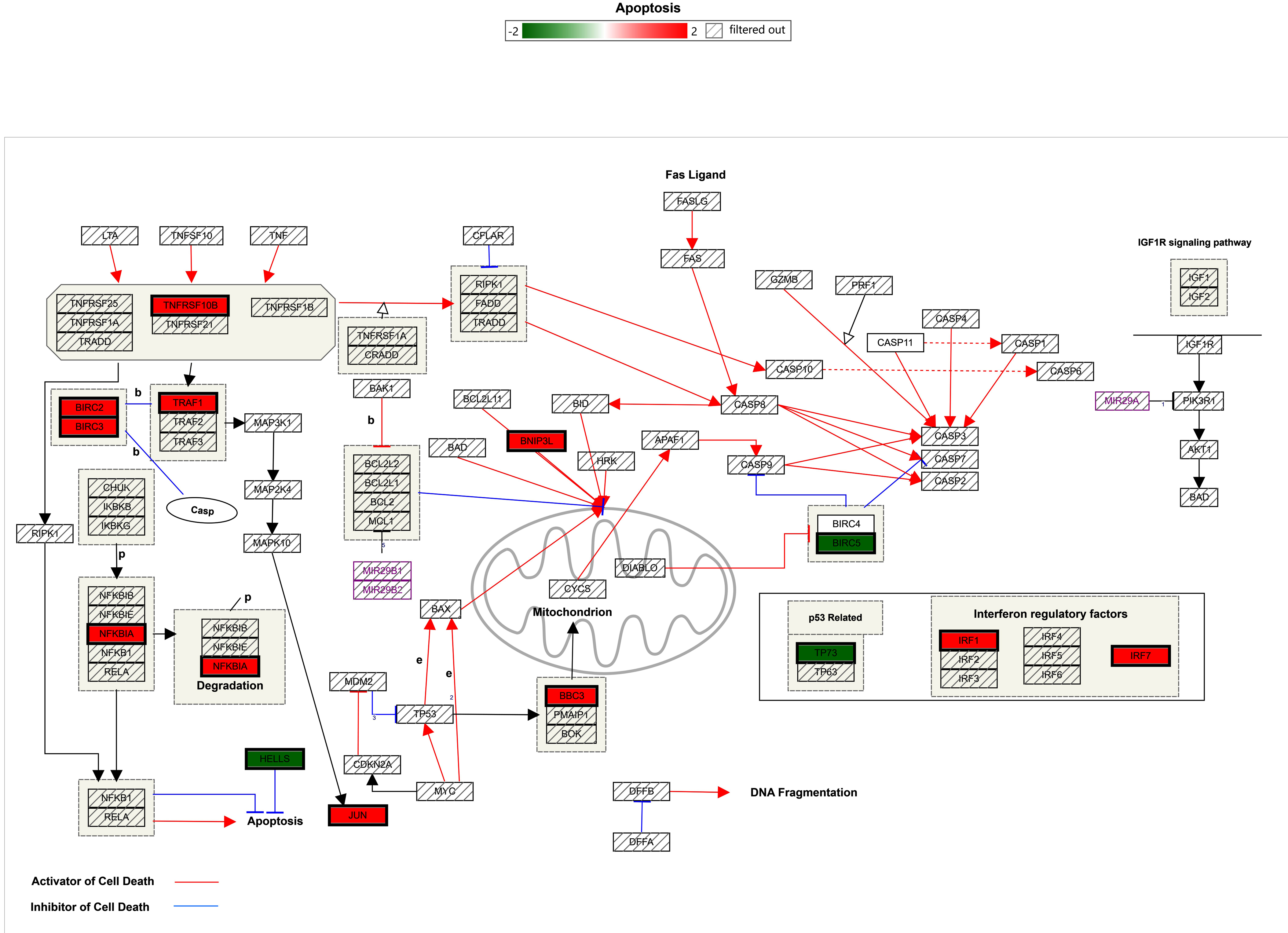

Supplement: Figure S10 — The figure shows component genes in AP-WP that were differentially regulated by MP-HX in HCT116 cells. The genes in the pathway are colored red (MA_FC ≥ + 2.0), or green (MA_FC ≥ − 2.0), or depicted as grey hashed boxes (MA_FC < ±2). [file peerj-06-5203-s012.jpg]

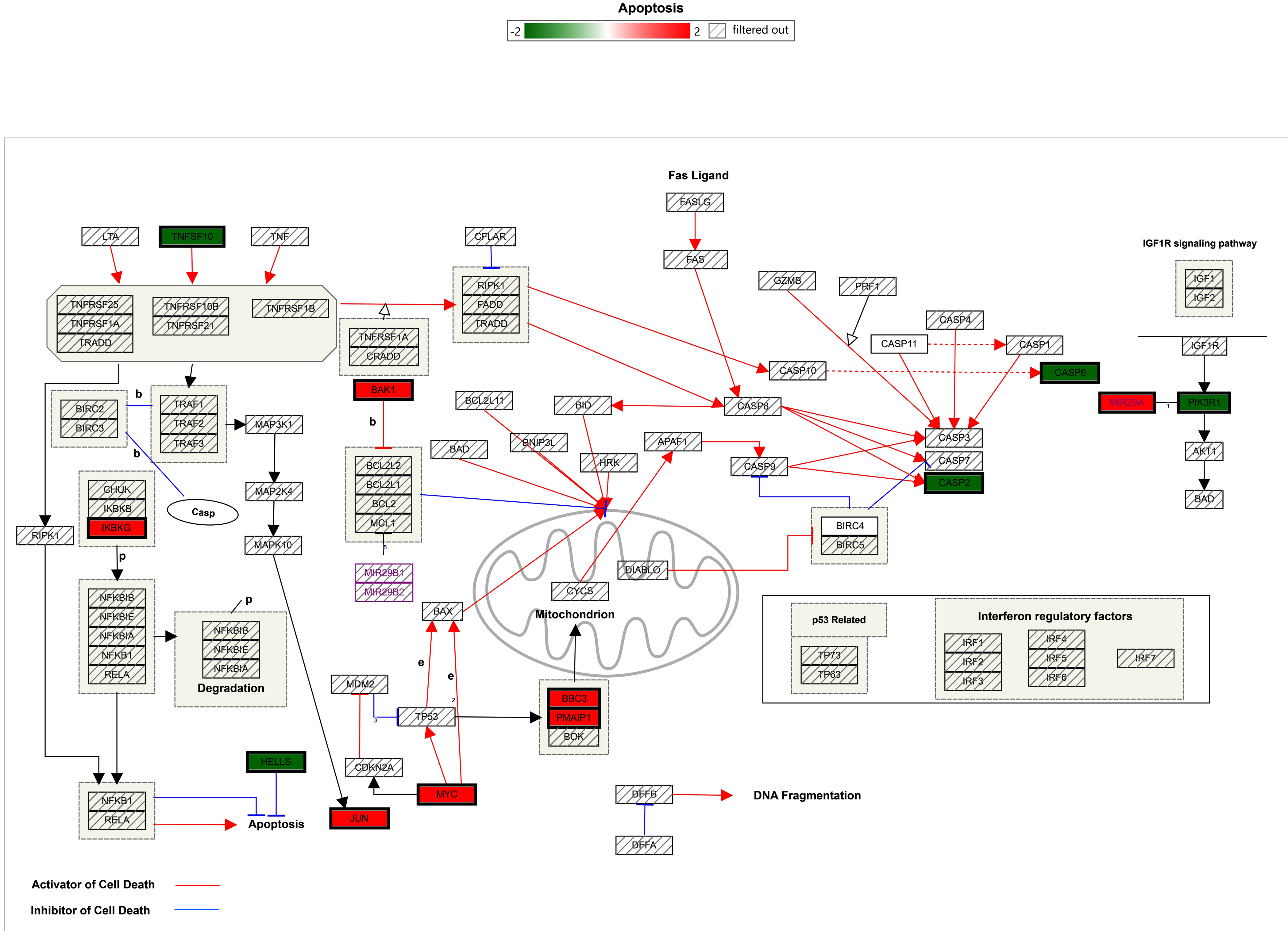

Supplement: Figure S11 — The figure shows component genes in AP-WP that were differentially regulated (MA_FC ≥ ±2.00) by MP-HX in HepG2 cells. The genes in the pathway are colored red (MA_FC ≥ + 2.0), or green (MA_FC ≥ − 2.0), or grey (MA_FC < ±2). [file peerj-06-5203-s013.jpg]

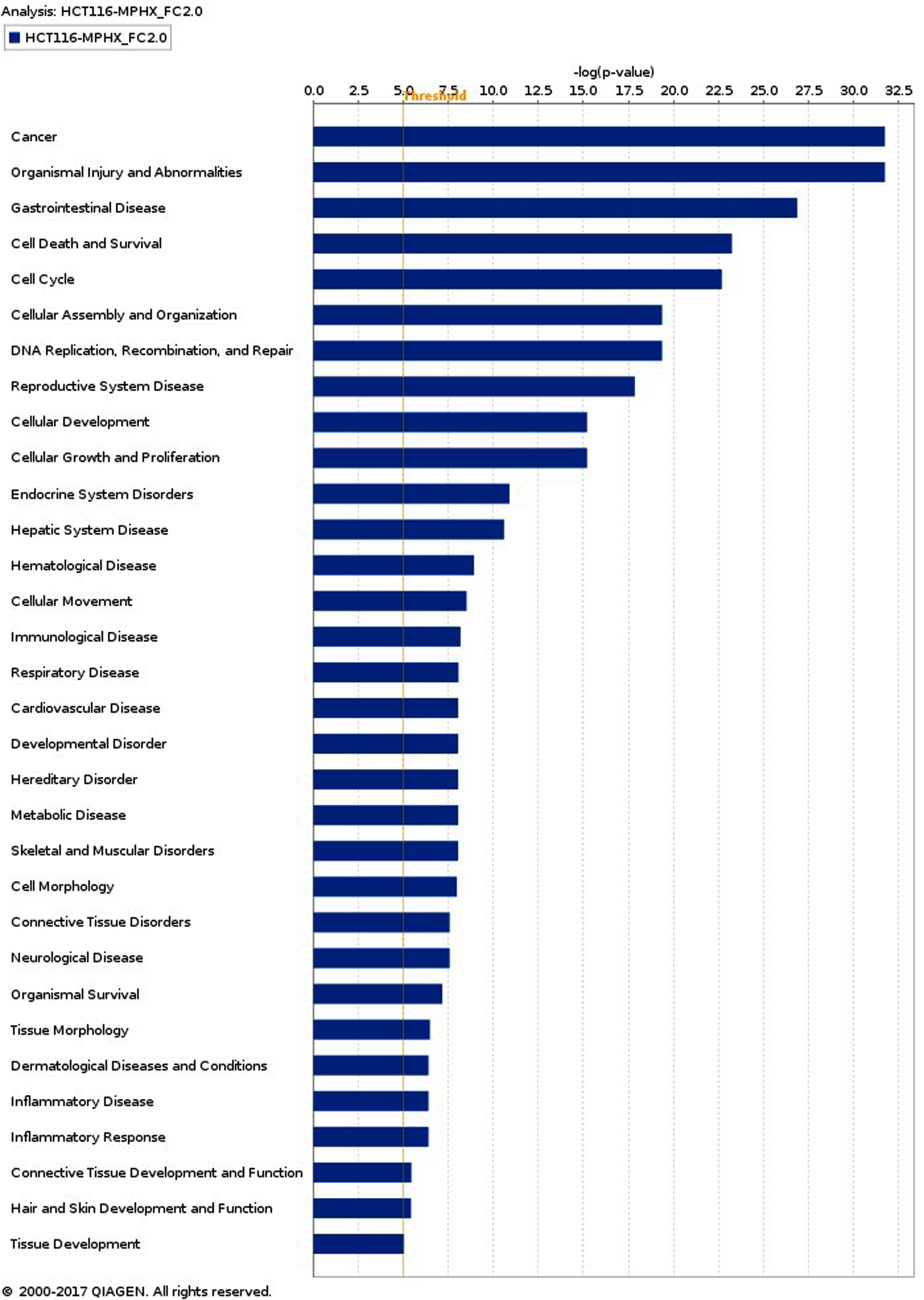

Supplement: Figure S12 — The figure shows top category of diseases and biological functions that were modulated by MP-HX (FC ≥ ±2.0) in HCT116 cells, and they were ranked by IPA software based on -log (p-value) ≥5.0. [file peerj-06-5203-s014.png]

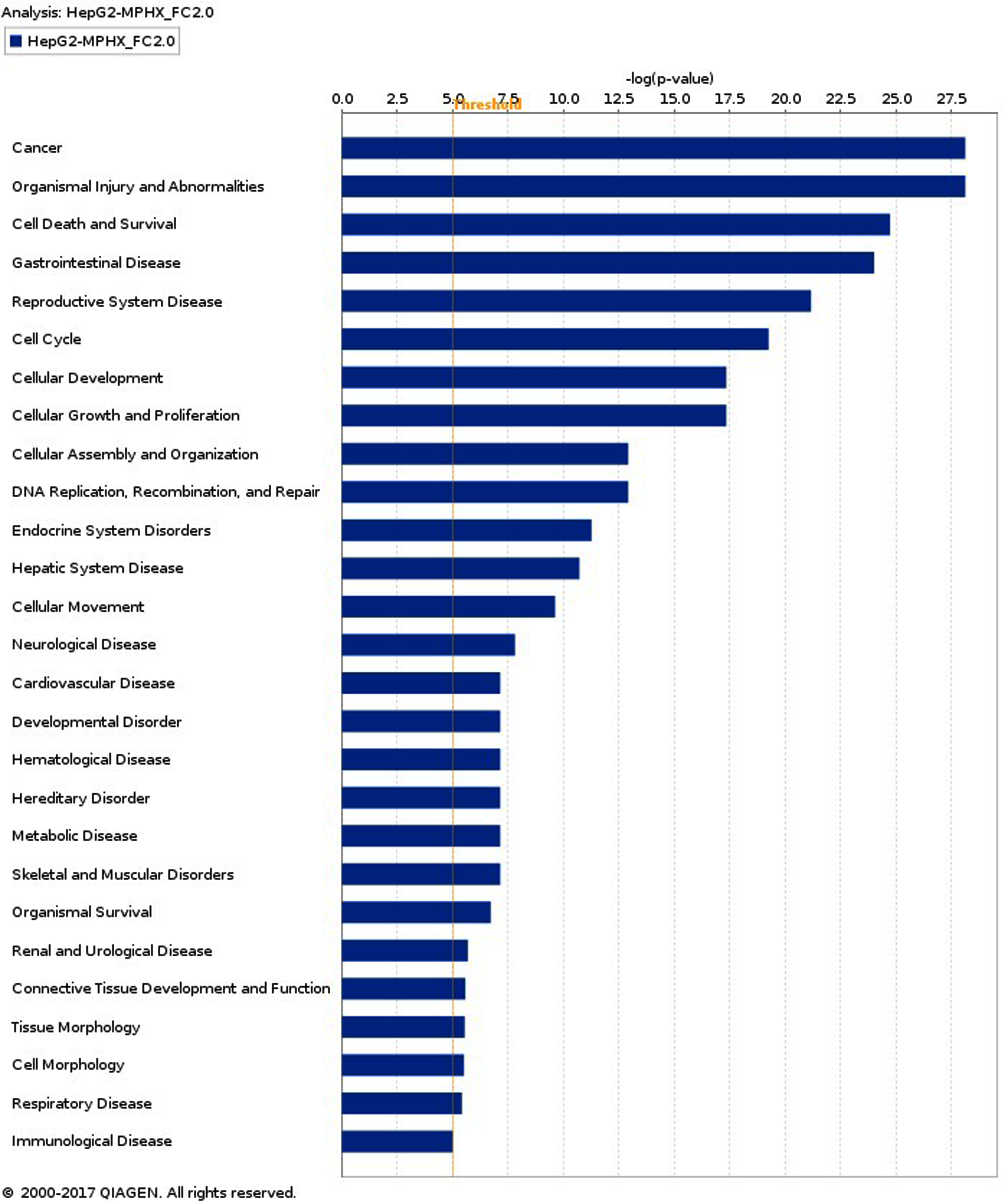

Supplement: Figure S13 — The figure shows top category of diseases and biological functions that were modulated by MP-HX (FC ≥ ±2.0) in HepG2 cells and they were ranked by IPA software based on -log (p-value) ≥5.0. [file peerj-06-5203-s015.png]
